# Transcriptomic and biochemical analyses reveal wheat drought mitigation by *Trichoderma simmonsii* and reduced demand for canonical plant stress responses

**DOI:** 10.3389/fpls.2025.1716657

**Published:** 2025-11-17

**Authors:** Julio Ascaso, David Mendoza-Salido, Alberto Pedrero-Méndez, Enrique Monte, Narciso M. Quijada, Rosa Hermosa

**Affiliations:** Department of Microbiology and Genetics, Institute for Agribiotechnology Research (CIALE), University of Salamanca, Villamayor, Spain

**Keywords:** water stress, ROS scavenging, Triticum aestivum, photosynthesis, transcription factors

## Abstract

**Introduction:**

Drought negatively affects production in wheat, an important crop worldwide. Some *Trichoderma* spp. are of interest for sustainable agriculture. Upon plant root colonization, some strains produce multifaceted benefits for its host, including defence priming against environmental stresses.

**Methods:**

Here, we investigated the physiological and biochemical responses of wheat (*Triticum aestivum* L. cv. Basilio) plants to *Trichoderma asperellum* T25 and *T. simmonsii* T137 treatments, applied by mycelium inoculation to plant growth substrate, and subjected to optimal irrigation, water stress (WS), and recovery upon rehydration. WS consisted in removing irrigation for 10 days in 14-day-old plants, and rehydration was performed by optimal irrigation for three days. RNA-sequencing analysis was performed on 24-day-old plants inoculated with T137, under different irrigation conditions, using uninoculated plants as controls.

**Results and discussion:**

Rubisco genes were upregulated in *Trichoderma*-inoculated plants in comparison with those untreated, independently of the irrigation condition. Under WS, 1,913 differentially expressed genes (DEGs), many of them involved in pathways related to plant stress responses, were associated with the *Trichoderma* application. Carbohydrate metabolism and photosynthesis were the main functional categories overrepresented of upregulated DEGs when comparing *Trichoderma*-WS and control-WS plants. Such upregulation was accompanied by downregulation of genes involved in biosynthesis of abscisic acid and osmolytes like proline and trehalose, and non-enzymatic antioxidants, in WS *Trichoderma*-treated plants. Those results together with a healthy phenotype and reduced hydrogen peroxide, proline and malondialdehyde levels indicate a minimal activation of the WS response in *Trichoderma*-treated plants. We detected 57 wheat transcription factor genes differentially expressed between *Trichoderma*-WS and control-WS treatments, with overrepresented members of WRKY, MYB, bHLH, NAC and C2H2 families. Our findings provide valuable insights on the protective effect of *Trichoderma* in wheat plants against drought, an environmental scenario that is increasing with global warming.

## Introduction

Current agriculture faces the need to ensure food for the global growing population, which is expected to reach 9 billion by 2050 (FAO, 2024), in a scenario where heating and drought are increasing with global warming (Rockström et al., 2023). In this context, wheat has a pivotal role since the 2023/24 predicted sowing and production figures were 222.2 million ha and 780 million tons, respectively (FAO, 2024), showing it as one of the most important crops worldwide. Drought is considered a major abiotic factor adversely affecting crop productivity (Fahad et al., 2017). Wheat exhibits high sensitivity to drought (Zampieri et al., 2017), with yield losses of up to 20% (Daryanto et al., 2016). Plants have developed specific adaptive features including physiological, biochemical, and molecular responses to cope with adverse conditions (Boyer, 1982; Farooq et al., 2009). Reduction of leaf water potential, stomatal conductance, photosynthesis net, and growth rates are plant responses associated to water scarcity (Farooq et al., 2009). Moreover, plants activate enzymatic and non-enzymatic ROS-scavenging systems to reduce the oxidative damage provoked by drought (Bandurska, 2022). Other known plant responses to drought are accumulation of ABA and specific proteins such as LEA and dehydrins (Sharma et al., 2019; Bandurska, 2022). In addition to ABA, other phytohormones are involved in plant growth and development, and are crucial for abiotic and biotic stress responses (Mittler and Blumwald, 2015; Verma et al., 2016; Iqbal et al., 2022). In addition, plant recovery capacity by rehydration after a long period of water deficit can reduce the damage and the impact on plant production, although studies on recovery upon rehydration are scarce (Abid et al., 2018).

*Trichoderma* is a filamentous fungal genus including species that are explored as an environmentally friendly alternative to agrochemicals increase crop yields (Woo et al., 2023). In addition to the antagonistic properties against pathogens of agricultural interest, some *Trichoderma* strains can improve plant growth and induce systemic tolerance to biotic and abiotic stresses (Harman et al., 2004; Hermosa et al., 2012). A pioneer work showed that *Trichoderma* spp. is able to activate the antioxidant machinery of tomato seedlings and their resistance to water deficit (Mastouri et al., 2012). However, studies analysing the responses to water stress (WS) of wheat plants treated with *Trichoderma* are relatively limited and recent. We have recently reported that *Trichoderma* spp. are able to reduce oxidative levels in wheat plants subjected to WS by increasing SOD, POX and CAT activities (Pedrero-Méndez et al., 2021; Illescas et al., 2022). *Trichoderma* application can also modify antioxidant metabolite levels such as proline (Illescas et al., 2022), provide phytohormones (Illescas et al., 2021), and decrease the ET substrate 1-aminocyclopropane-1-carboxylic acid (ACC) levels in wheat plants subjected to abiotic stresses (Zhang et al., 2019; Illescas et al., 2021; Rauf et al., 2021; Illescas et al., 2022). Strains from different *Trichoderma* spp. and using different application ways such as *Trichoderma asperellum* applied by seed coating (Illescas et al., 2022) and *Trichoderma harzianum* applied as seed-biopriming and foliar spray (Aljeddani et al., 2024), showed ability to protect wheat plants against drought. Particularly, *Trichoderma simmonsii* T137, a strain isolated from root endosphere of healthy wheat plants and applied to the plant growth substrate, was selected on the basis of its ability to increase wheat tolerance to severe WS (Pedrero-Méndez et al., 2021).

RNA-sequencing (RNA-Seq) analysis performed in rice and tomato plants have served to associate plant tolerance to WS with the transcriptomic changes caused by *Trichoderma* treatments (Bashyal et al., 2021; De Palma et al., 2021). However, due to the complexity of the wheat genome, the functional diversity of homeologs and the still uncleared regulatory pathways of phytohormones (IWGSC, 2018), the gene networks that regulate plant beneficial traits triggered by *Trichoderma* under adverse conditions remain poorly explored (Illescas et al., 2022; Aljeddani et al., 2024).

In this study, greenhouse assays and RNA-Seq analysis have served to explore the effects of *Trichoderma* on the tolerance of wheat plants to WS and their recovery after rehydration. To this aim, we first measured physiological and biochemical parameters in wheat plants from different treatments and irrigation conditions: *Trichoderma* application (uninoculated, *T. asperellum* T25, *T. simmonsii* T137) and water regimes [optimal irrigation (OI), WS, recovery upon rehydration]. Thus, using RNA-Seq analysis, we identified transcriptomic changes associated with the improved responses of T137-treated plants to WS and their recovery upon rehydration.

## Materials and methods

### Plant material, *Trichoderma* inoculation and experimental design of greenhouse assays

Winter wheat (*Triticum aestivum* L. cv. Basilio) seeds, provided by ACOR Cooperative (Valladolid, Spain), were surface-disinfected by rinsing in 1% sodium hypochlorite for 10 min, washed once with sterile water, soaked in HCl 0.1 N for 1 min, and then washed four times with sterile water. The seeds were cold stratified at 4°C for three days to promote germination. The plant growth substrate consisted of a twice autoclaved mix of commercial peat (Projar Professional, Comercial Projar SA, Fuente el Saz de Jarama, Spain) and vermiculite (3:1) (v/v). *T. asperellum* CECT 20178 (Spanish Type Culture Collection, Valencia, Spain), referred to as T25, and *T. simmonsii* T137 (Pedrero-Méndez et al., 2021) strains were utilized in two experimental trials, and fungal mycelium was used to inoculate the plant growth substrate. Mycelium was obtained from a potato dextrose broth (PDB, Difco Laboratories, Detroit, MI, USA) culture, using 0.5 L flasks containing 250 mL of medium inoculated with 1 x 10^6^ conidia/mL, and grown at 180 rpm and 28°C for 4 days. Mycelium was collected by filtration and washed with sterile water to remove the medium completely. Ten grams of fungal mycelium were resuspended in 6 L of water, mixed with the plant-growth substrate and used to fill 42 pots of 125 mL (pot size: 5.5 cm diameter, 17 cm height). A total of 126 pots were used. T25-inoculated, T137-inoculated and non-inoculated (control) pots were sowed with two seeds per pot, and seedlings were grown in a greenhouse at 22 ± 4°C, 60% relative humidity, 16 h light/8 h dark photoperiod, and watered regularly to maintain substrate moisture close to 100% field capacity for two weeks. After 14 days, when seedlings had completely formed the fourth leaf, potted plants from the three batches (control, T25, T137) were separated into two groups: one group was used to evaluate the response to WS (WS experiment), and the second group was used to study recovery upon rehydration (recovery experiment). A complete randomized design was adopted for both experiments, and three trays with seven replicates were assigned for each treatment.

For the WS experiment, the following six experimental treatments were considered: non-inoculated (C-OI), T25-inoculated (T25-OI) and T137-inoculated (T137-OI) plants maintained in OI condition; non-inoculated (C-WS), T25-inoculated (T25-WS) and T137-inoculated (T137-WS) water-stressed plants. WS consisted of completely preventing irrigation for ten days. When plants were 24 days old, three random biological replicates (seven pots and two plants per pot for each replicate) were separately collected for each treatment. For each biological replicate: aerial parts of plants from four pots were collected and used to determine fresh and dry weights, and RWC; and plant leaves from three pots were harvested, immediately frozen in liquid nitrogen, and kept at -80°C for biochemical and transcriptomic analyses. Samples T25-OI and T25-WS were not included in the transcriptomic analysis.

For the recovery experiment, the following six experimental treatments were considered: non-inoculated (C-OI+OI), T25-inoculated (T25-OI+OI) and T137-inoculated (T137-OI+OI) plants maintained under OI condition; and non-inoculated (C-WS+OI), T25-inoculated (T25-WS+OI) and T137-inoculated (T137-WS+OI) water-stressed and rehydrated plants. The WS plus rehydration condition consisted of removing irrigation for 10 days with subsequent rehydration for three days. Thus, on 27-day-old plants, samples were collected and intended for analyses as indicated above. Samples T25-OI+OI and T25-WS+OI were not included in the transcriptomic analysis.

### Physiological parameters

At sampling time, seedling shoots were split to determine FW and then oven-dried at 80 °C for 4 days until weight was constant to obtain DW. The RWC was measured on the last fully expanded wheat leaf using 1 cm segments, and following methodology previously reported (Teulat et al., 2003).

### Biochemical measurements

Hydrogen peroxide (H_2_O_2_) content was determined in wheat shoots according to Velikova et al. (2000), and using 50 mg of frozen plant material. The absorbance of supernatant was determined in a spectrophotometer at 390 nm, and H_2_O_2_ content was calculated according to a standard curve. Results are expressed in micromoles (μm) of H_2_O_2_/g FW. MDA level was measured as previously described (Quan et al., 2004; Illescas et al., 2022), and expressed as μmol of MDA/g FW. Proline content was determined by the ninhydrin method (Bates et al., 1973), with modifications as previously reported (Illescas et al., 2022). Proline concentration was determined from a standard curve, and results expressed as μmol of proline/g FW. Measurements of H_2_O_2_, MDA and proline were carried out with three technical replicates for each of the three biological replicates.

Antioxidant enzymatic activities were evaluated as previously described (Nawaz et al., 2020; Illescas et al., 2021). CAT and POX enzyme units were defined as the increase of 0.1 absorbance value per min; and one unit of SOD was considered as the amount of enzyme needed to cause 50% inhibition in the photochemical reduction of Nitro blue tetrazolium chloride (Thermo Fisher Scientific, Walthman, MA, USA). Protein concentration was determined using Pierce^TM^ Bradford Plus Protein Assay Reagent (Thermo Fisher Scientific) and bovine serum albumin as standard protein. Antioxidant activities were performed with three technical replicates for each of the three biological replicates.

### RNA library preparation and sequencing

Total RNA was extracted from 100 mg of wheat leaves using TRIzol reagent (Thermo Fisher Scientific), and treated with Ambion RNase-free DNase I (Thermo Fisher Scientific) according to the manufacturer’s protocol. RNA quantification was analysed by a Qubit fluorometer (Thermo Fisher Scientific), and its quality was checked on a 2100 Bioanalyzer platform (Agilent Technologies, Santa Clara, CA, USA). Three replicates for each treatment were used, and each replicate included a leaf pool taken of the six plants from three pots. In total, 24 libraries were constructed in this study: three biological replicates for each of the four treatments for the WS experiment (C-OI, T137-OI, C-WS and T137-WS), and three biological replicates for each of the four treatments for the recovery experiment (C-OI+OI, T137-OI+OI, C-WS+OI and T137-WS+OI).

Library preparation and sequencing were carried out by Fundación Parque Científico de Madrid (Madrid, Spain). Sequence libraries were prepared using a TruSeq RNA Sample Preparation Kit v2 (Illumina, San Diego, CA, USA) and pooled in equimolar concentration. Sequence libraries were purified and concentrated using Agencourt AMPure XP (Thermo Fisher Scientific) and sequenced on an Illumina NovaSeq SP (200 cycles) yielding a median of 72.5 million 100 bp paired end reads per sample. Raw data for all treatments from RNA-Seq have been deposited in the NCBI Sequence Read Archive (SRA), Bioproject PRJNA1259720 (24 mRNA wheat libraries, run numbers SAMN48371003 to SAMN48371026).

### Bioinformatic analysis of the RNA-Seq data

The quality of the raw sequencing data was evaluated by using FastQC (Andrew, 2010) and MultiQC (Ewels et al., 2016). PolyA tail adapters were removed by using Cutadapt (Martin, 2011) and enabling the “–poly-a” option. Other residual barcodes and adapters were removed using Trimmomatic v.0.39 (Bolger et al., 2014). Sequences with a mean Phread quality score below 30 and a length shorter than 70 nucleotides were discarded using FastP v.0.23.4 (Chen, 2023). Potential Phi-X174 and human DNA residues were removed using Bowtie2 v.2.5.1 (Langmead et al., 2009).

The *T. aestivum* reference genome version 2.1 was downloaded from its public repository (IWGSC RefSeq v2.1, https://www.wheatgenome.org/projects/reference-genome-project/refseq-v2.1; accessed on May 8, 2024). The quality-controlled sequencing data were aligned against the wheat reference genome using HISAT2 v.2.2.1 (Pertea et al., 2016). Transcripts' coverages were estimated by using StringTie v.2.2.1, and read counts per transcript were calculated. With the count of reads mapped to each gene, Transcripts Per Kilobase Million (TPM) were calculated using the formula (Zhao et al., 2021): TPM = (Gene Count / Gene Length) / Σ (Gene Count / Gene Length) x 10^6^.

The high confidence genes from the wheat reference genome (IWGSC RefSeq v2.1) were functional annotated with the KEGG (Kanehisa et al., 2023); accessed on November 6, 2024, and using the sequence aligner Diamond v.2.1.8 (Buchfink et al., 2021). Annotations were considered when the sequence alignment to each protein overcame a minimum of 70% coverage and identity. Moreover, the list of wheat genes encoding putative TFs, previously reported (Chen et al., 2023), was used in this study.

Additionally, several GO terms were manually selected and used to develop a custom database for further screening of the *T. aesticum* genome. The GO terms belonging to the biological process’s classification were searched: response to ABA (GO:0009737), response to JA (GO:0009753), response to SA (GO:0009751), response to water deprivation (GO:0009414) and water transport (GO:0006833).

#### Differential gene expression analysis

The pairwise differential gene expression analysis between the different treatments (three biological replicates per treatment) was performed using DESeq2 v.1.44.0 (Love et al., 2014). Only those genes with TPM >= 50 across all samples were included in the differential gene expression analysis. The resulting *p* values were adjusted using the Benjamini and Hochberg’s method to decrease the false discovery rate (FDR). Differentially Expressed Genes (DEGs) were considered when an adjusted *p* value < 0.05 and a |log_2_ Fold Change*|* > 1 were found between treatments.

#### Weighted co-expression network analysis

With the aim of detecting possible gene regulatory networks and key regulators, known as hub genes, we performed a weighted gene co-expression network analysis (WGCNA). From the normalized TPM gene matrix, genes with low expression were removed (greater than 3 TPM for at least three treatments), as well as confounding artifacts in order to avoid false-positive correlations. For the gene co-expression network inference, we used the package BioNERO (Almeida-Silva and Venancio, 2022), setting the net type in “signed hybrid” so we only considered positive correlations. The value used to generate a scale-free topology network (networks with a non-random connectivity) was 15. After module detection, those modules with a similar correlation of their eigengenes were merged. We selected a module based on its connection with environmental adaptation and the high frequency of relevant TFs, and only genes with a correlation higher than 0.8 were selected for visualization.

### RNA-Seq validation by quantitative real-time PCR

DNase-treated samples from RNA libraries were used for validating the expression profiles of some of the key genes and TFs for abiotic plant responses by quantitative real-time PCR (qPCR). cDNA was synthesized from 1 µg of RNA using PrimeScript™ RT reagent Kit (Takara, Inc., Tokyo, Japan) following the manufacturer’s instructions. PCR reactions were performed on a StepOnePlus thermocycler (Applied Biosystems, Foster City, CA, USA) and using SYBR FAST KAPA qPCR (Biosystems, Buenos Aires, Argentina) as previously described (Rubio et al., 2019). The expression levels of genes *WZY2* (TraesCS6A03G0901500) encoding a dehydrin, *DHN16* (TraesCS6B03G1084000) for a LEA protein, *P5CR* (TraesCS3B03G1339500) for a pyrroline-5-carboxylate reductase, *LOX1* (TraesCS4B03G0082800) for lipoxygenase 1, and *GRAS* (TraesCS2B03G0579900), *WRKY* (TraesCS6B03G0446800), *NAC* (TraesCS3B03G0504300) and *ERF* (TraesCS2B03G1138900), for TFs, were analysed. Primer sequences designed in this study, or previously reported (Rubio et al., 2019; Illescas et al., 2022; Risoli et al., 2023), are listed in **Supplementary Table S1**. For primer design, the sequences of the three homoeologous copies of each target gene were retrieved from the wheat genome assembly IWGSC v2.1 available at Phytozome (https://phytozome-next.jgi.doe.gov/; accessed on June 10, 2025). The sequences were aligned using Clustal Omega (Sievers et al., 2011), and alignments were visually inspected to identify single-nucleotide polymorphisms (SNPs) suitable for homoeolog-specific primer design. Primers were then manually designed to selectively amplify a single homoeolog based on these diagnostic SNPs. Ct values were normalized with the *ACTIN* gene, and expression levels were calculated according to the 2^-ΔΔCt^ method (Livak and Schmittgen, 2001).

### Statistical analysis of physiological and biochemical data

Plant variables (collected from at least three biological replicates) were subjected to analysis of variance (ANOVA) with ‘*Trichoderma* inoculation’ and ‘irrigation condition’ as the main factors, including the interaction between variables. Data homoscedasticity and normality were checked with Levene’s test and Shapiro-Wilk’s test before statistical analysis. Tukey’s HSD *post hoc* test (ns, not significant; *p < 0.05; ** *p* < 0.01; *** *p* < 0.001) was used in multiple-difference comparisons. All statistical analyses of plant variables were performed by using dplyr, stat, tidyr, car and multcomp packages under R environment version 4.3.3.

### Data visualization

Visualization was performed by using the ggplot2 package (Wickham et al., 2016) in R environment. Venn diagrams and Upset plots served to spot shared DEGs between conditions using VennDiagram (Chen and Boutros, 2011) and ComplexUpset Krassowski packages. A logarithmic transformation “log(1+x)” of the TPM values was used to reduce the disproportionate contribution of the most expressed genes, in comparison with the lowest ones, and the Rtsne package (van der Maaten and Hinton, 2008) served to perform t-SNE (t-distributed stochastic neighbor embedding). The ggkegg package (Sato et al., 2023) was used to represent the proline and ethylene biosynthesis pathways. A KEGG enrichment analysis was carried out to obtain the differentially expressed KEGGs between treatments; and by using log_2_ FoldChange, the presence or absence of the KEGGs by their behaviour were plotted.

## Results

### *T. asperellum* and *T. simmonsii* application positively affects wheat plants subjected to water stress and recovery upon rehydration

**Figure 1:**
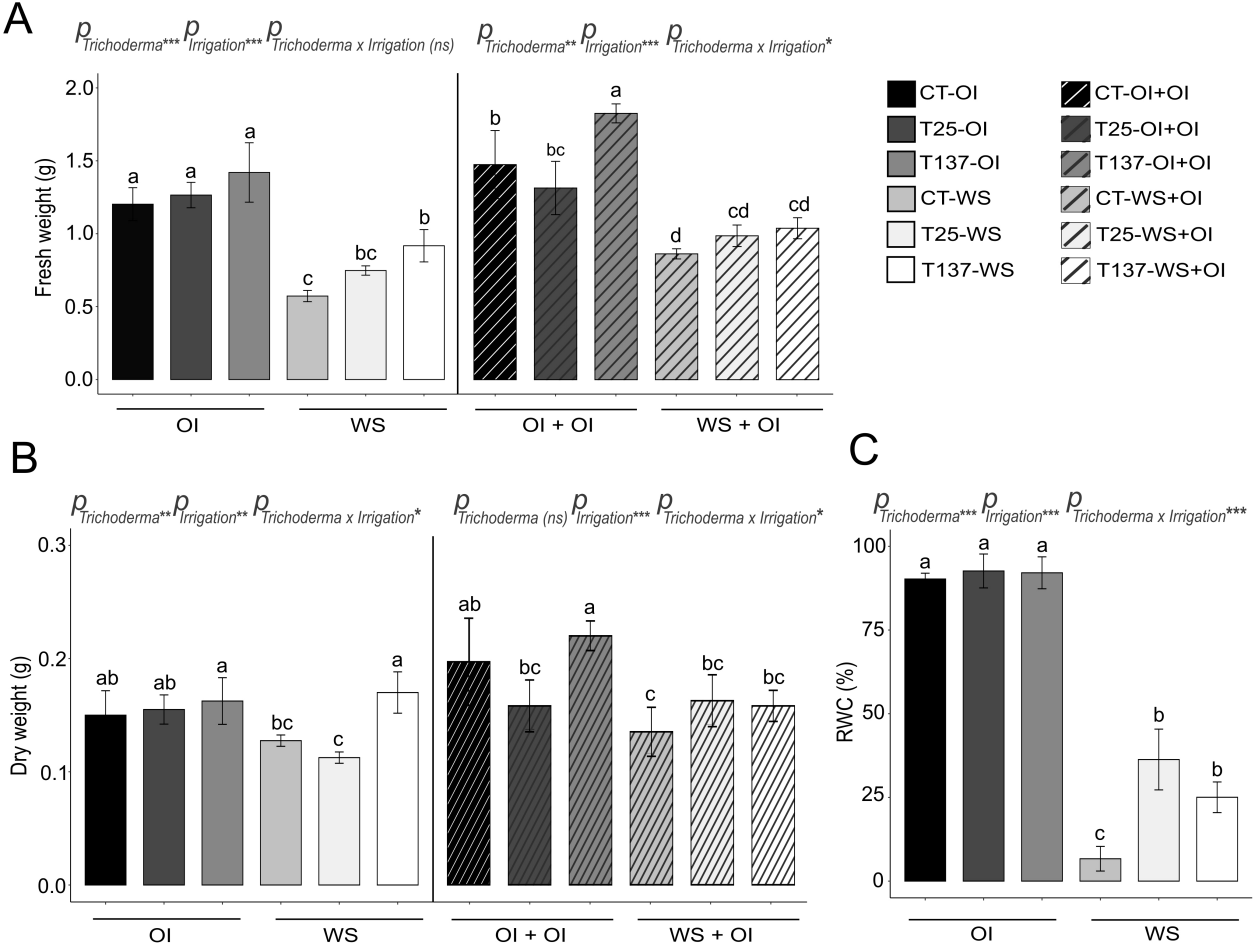
Impact of water stress and rehydration on physiological measurements of *Trichoderma*-treated and untreated wheat plants. Fresh and dry weights of non-stressed, water-stressed and rehydrated plants **(A, B)**. Relative water content (RWC) of non-stressed and water-stressed plants **(C)**. Values are means ± standard deviation of three independent biological replicates, different letters indicate significant differences (Tukey’s test, *p* < 0.05). OI: Optimal Irrigation. WS: Water stress. OI+OI: Optimal irrigation. WS+OI: Rehydration after WS. For each set of data, significance effect was determined by two-way ANOVA for *Trichoderma* treatment, irrigation condition and their combination (***: *p* < 0.001; **: *p* < 0.01; * *p* < 0.05; ns: no statistical differences).

**Figure 2:**
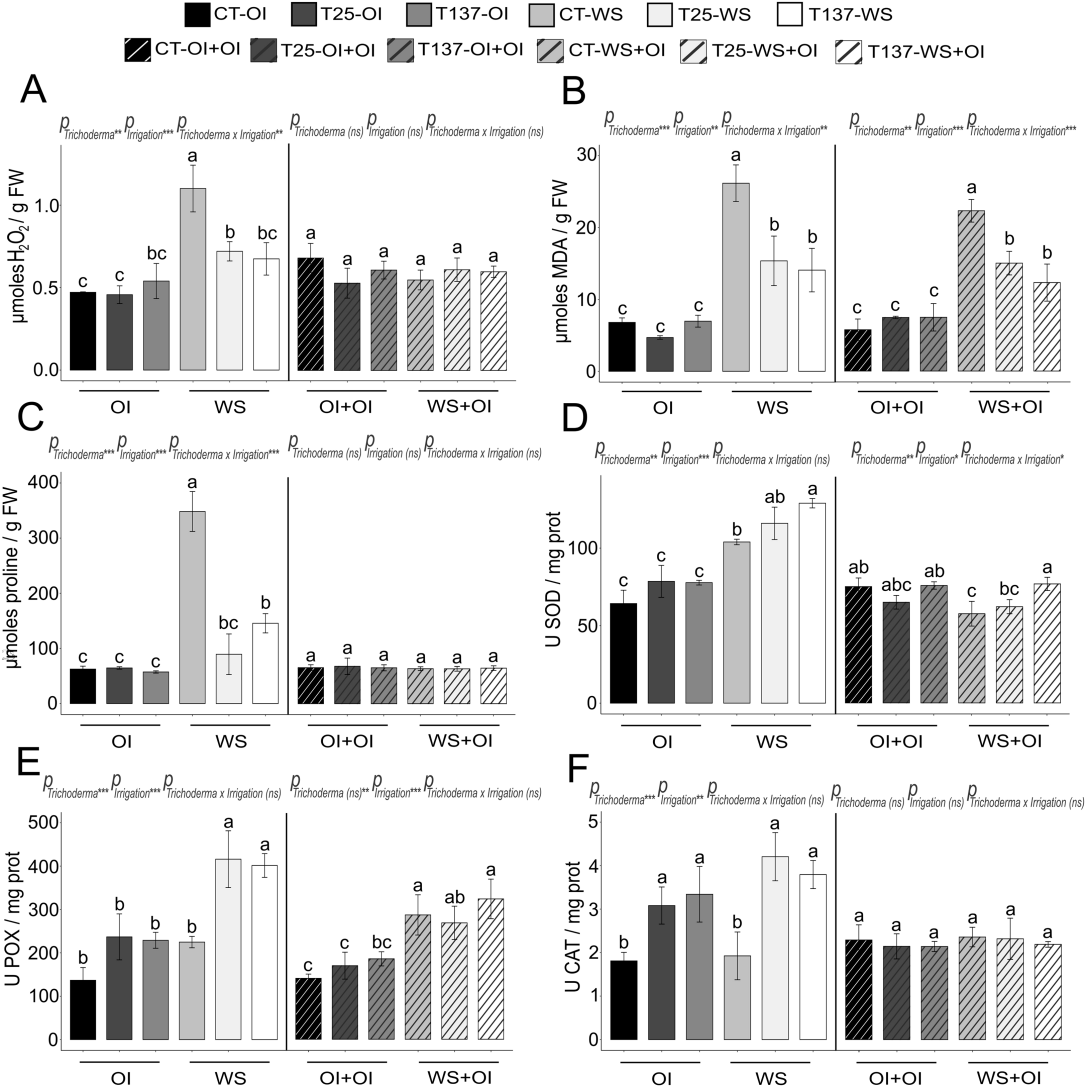
Impact of water stress and rehydration on biochemical measurements of *Trichoderma*-treated and untreated wheat plants. Hydrogen peroxide (H_2_O_2_), proline and malondialdehyde (MDA) contents of non-stressed, water-stressed and rehydrated plants **(A–C)**. Superoxide dismutase (SOD), peroxidase (POX) and catalase (CAT) activities of non-stressed, water-stressed and rehydrated plants **(D–F)**. Values are means ± standard deviation of three independent biological replicates, different letters indicate significant differences (Tukey’s test, *p* < 0.05). OI: Optimal Irrigation. WS: Water stress. OI+OI: Optimal irrigation. WS+OI: Rehydration after WS. For each set of data, significance effect was determined by two-way ANOVA for *Trichoderma* treatment, irrigation condition and their combination (***: *p* < 0.001; **: *p* < 0.01; * *p* < 0.05; ns: no statistical differences).

To evaluate the ability of *Trichoderma* to protect wheat seedlings from drought stress, we firstly investigated the effect of *T. asperellum* T25 and *T. simmonsii* T137 on wheat seedlings under different irrigation conditions: OI, WS and recovery upon rehydration (**Figures 1** and **2**). Under the OI condition, *Trichoderma* inoculation did not alter substantially any investigated physiological (FW, DW and RWC) or biochemical (H_2_O_2_, proline, MDA, and antioxidant enzymatic activities) plant parameters in comparison to those of untreated plants. Notably, T137-treated plants showed the highest FW and DW in both OI and WS conditions, being statistically significant only for the T137-WS treatment (**Figure 1**). An increase of CAT activity was also observed in T25-OI and T137-OI plants compared to C-OI, although these differences were not maintained in T25-OI+OI and T137-OI+OI plants (**Figure 2**).

Water scarcity affects plant development, altering key metabolic processes that lead to oxidative stress. In this work, WS significantly affected all analysed physiological and biochemical parameters (**Figures 1** and **2**). WS plants showed wilting, leaf curling and less growth (**Supplementary Figure S1**), as well as lower RWC and higher oxidative damage compared to well-watered plants. FW and DW decreased under WS, and conversely, antioxidant enzymatic and non-enzymatic processes increased in WS plants. *Trichoderma* inoculation mitigated the effects of WS (**Supplementary Figure S1**). Results showed that *Trichoderma* application significantly increased leaf RWC in both T25-WS and T137-WS plants with respect to C-WS plants (**Figure 1**). However, only T137-inoculated plants showed higher FW and DW than uninoculated within the group of WS plants. Regarding biochemical measurements, WS increased H_2_O_2_, proline and lipid peroxidation levels in C-WS plants compared to those treated with *T. asperellum* T25 or *T. simmonsii* T137, indicating higher levels of oxidative damage in the non-inoculated plants (**Figure 2**). On the contrary, *Trichoderma* application enhanced the plant antioxidant enzymatic machinery under WS, increasing SOD, POX and CAT activities, and differences were not observed between plants of the treatments T25-WS and T137-WS. Except FW data, analysed physiological parameters were affected by the interaction of “Trichoderma” and “irrigation” factors (**Figure 1**).

Rehydration allowed the apparent plant recovery, as observed in the phenotype of rehydrated plants (**Supplementary Figure S1**), reducing the disturbances caused by the WS period. All rehydrated plants showed a decrease in FW compared to OI+OI plants, and DW differences were also observed between C-OI+OI and C-WS+OI and between T137-OI+OI and T137-WS+OI plants (**Figure 1**). No differences were observed in H_2_O_2_ and proline contents, and in SOD and CAT activities for rehydrated plants compared to those of OI+OI treatments (**Figure 2**). However, T137-WS+OI plants showed higher SOD activity levels than those of the C-WS+OI treatment. Although MDA levels were increased in plants of all rehydrated treatments with respect to OI+OI, *Trichoderma* inoculation provided lower MDA levels than those of the C-WS+OI treatment. Only MDA content and SOD activity levels were significantly affected by the interaction between *Trichoderma* and irrigation factors under rehydration (**Figure 2**).

### RNA-Seq data analysis and functional annotation of *T. simmonsii* T137-treated or untreated wheat plants in response to water stress and recovery upon rehydration

Wheat gene transcriptional profiles in response to *T. simmonsii* T137 application, WS and recovery upon rehydration were inferred by RNA-Seq. A total of 24 samples, three biological replicates for each of eight treatments, were subjected to RNA purification and further cDNA sequencing, yielding a total of 1.8 trillion reads (mean of 75 M sequences per sample) after quality control (**Table 1**). From them, approximately 91% of the reads aligned against CDS of the *T. aestivum* genome (version 2.1), and 55.57% was the portion of such reads that aligned against a CDS that harbors a functional annotation according to KEGG.

**Table 1:** Treatment code and description of the plant libraries sequenced in this study with indication of average reads after quality filtering and mapped to the reference wheat genome (version 2.1).

Treatment code	Description	Reads	Alignment in genome (%)
C-OI	Control optimally irrigated plants	67.5 ± 5.1	92.66
C-WS	Water-stressed control plants	85.9 ± 33.1	91.82
T137-OI	Optimally irrigated plants with *T. simmonsii* T137	70.1 ± 5.3	91.47
T137-WS	Water-stressed plants with *T. simmonsii* T137	82.3 ± 20.0	89.22
C-OI+OI	Control optimally irrigated plants + rehydration	79.8 ± 28.1	91.04
C-WS+OI	Water-stressed control plants + rehydration	67.7 ± 9.0	91.51
T137-OI+OI	Optimally irrigated plants with *T. simmonsii* T137 + rehydration	77.5 ± 8.9	87.94
T137-WS+OI	Water-stressed plants with *T. simmonsii* T137 + rehydration	69.4 ± 6.6	93.23

Read values are represented by millions.

**Figure 3:**
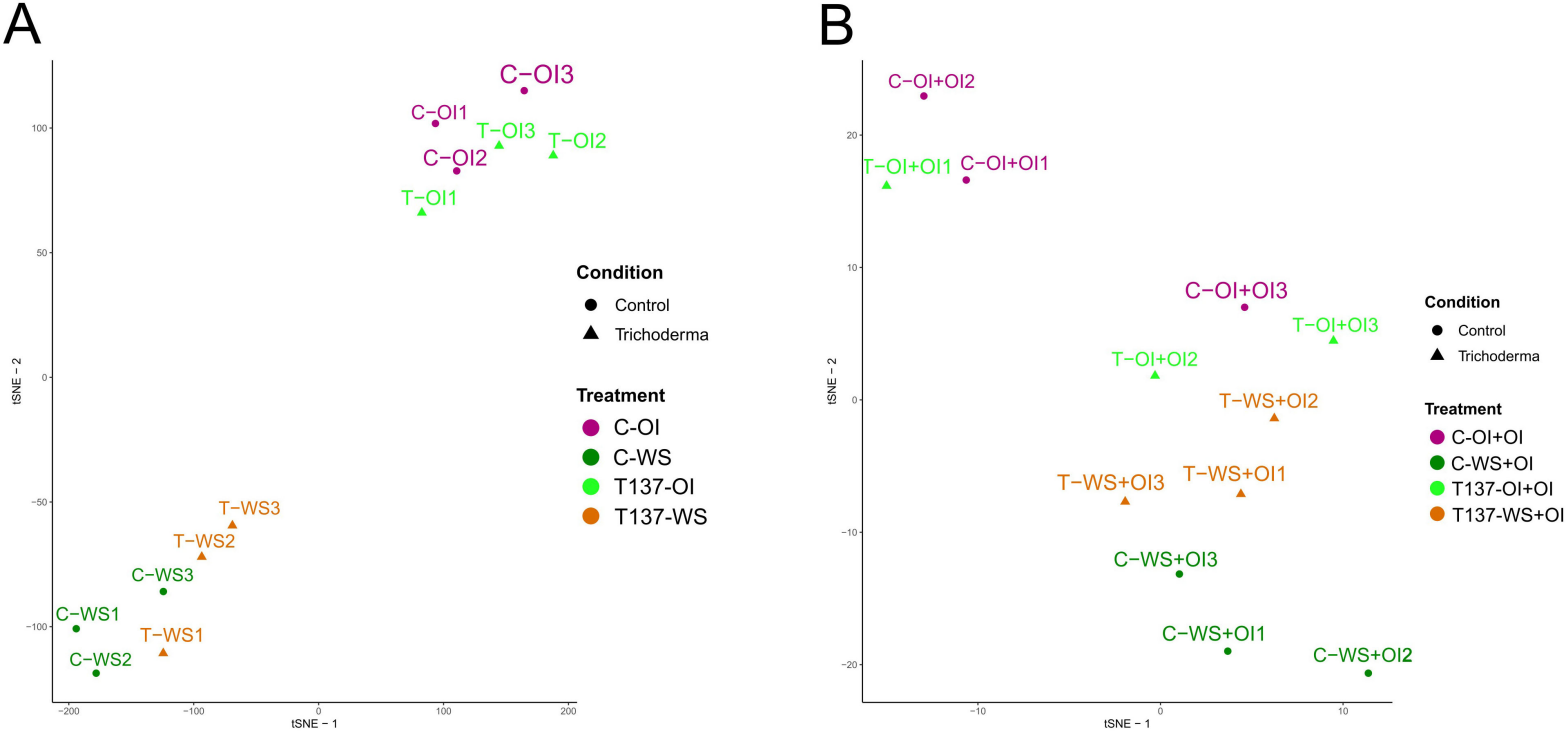
Overview of the wheat plant transcriptome, *Trichoderma*- treated (T137) and untreated (control) subjected to water stress (WS) and recovery (rehydration after WS) conditions using a T-distributed Stochastic Neighbour Embedding (tSNE). All biological samples from water stress (WS) experiment **(A)**. All biological samples from the recovery experiment **(B)**.

When samples only from the WS experiment were considered, t-SNE separately clustered those of WS and OI regardless of T137 application (**Figure 3A**). For the recovery experiment, t-SNE only grouped the C-WS+OI treatment samples separately, but placed the T137-WS+OI samples close to the non-water stressed samples (**Figure 3B**). WS was also the major impact factor influencing the t-SNE including all samples from WS and recovery experiments (**Supplementary Figure S2**); and C-WS and T137-WS samples were clustering aside, while the rest did not form clear groups.

**Figure 4:**
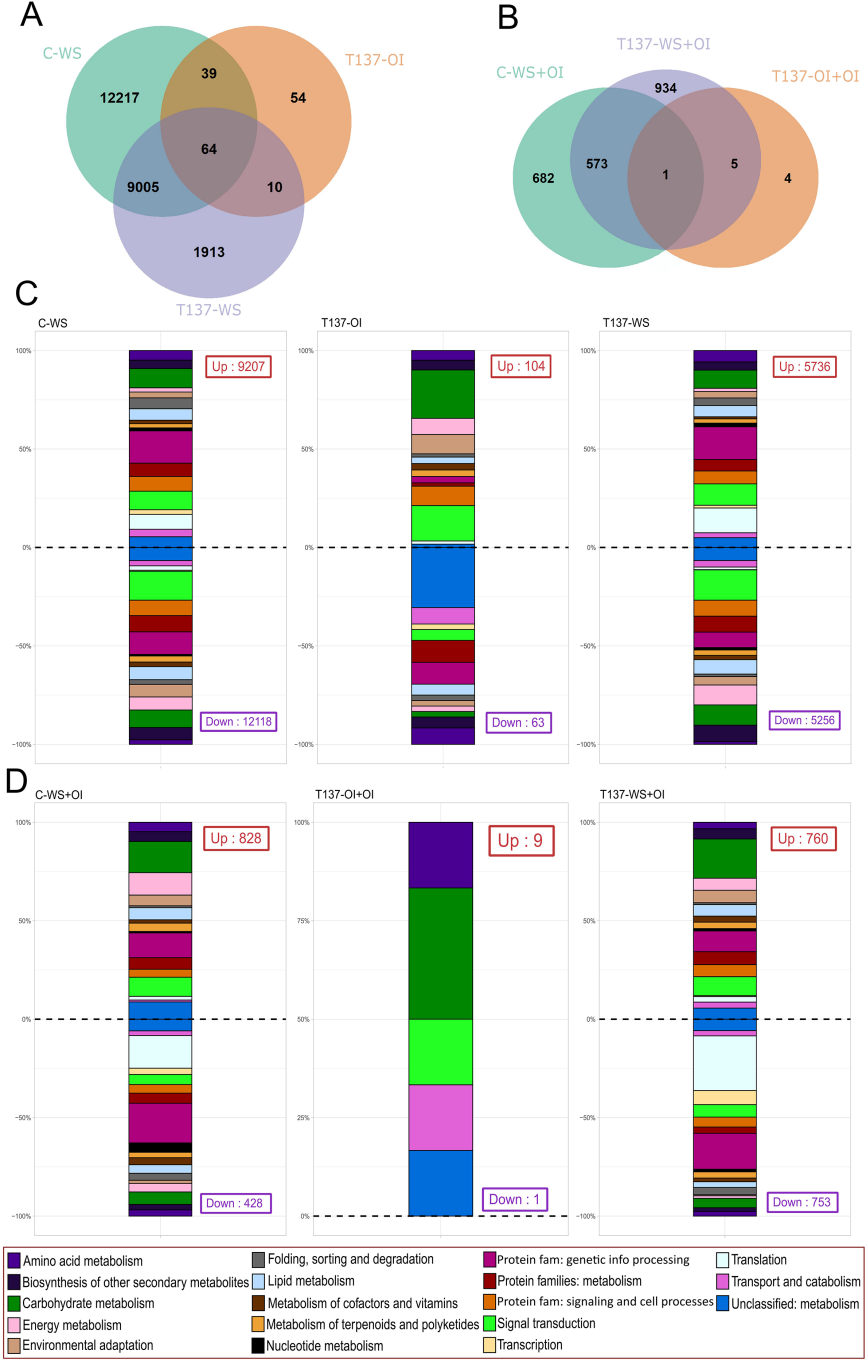
Differentially expressed genes (DEGs) between comparisons and their biological function using Kyoto Encyclopedia of Genes and Genomes (KEGG) annotation. Venn diagrams showing shared and unique DEGs of key comparisons against C-OI [water stress (WS) experiment] **(A)**, and against C-OI+OI (recovery experiment) **(B)**. Second level of KEGG annotation results of upregulated and downregulated DEGs obtained from WS and recovery experiments against their respective controls C-OI and C-OI+OI **(C, D)**.

After calculating DEGs between the different treatments against their respective controls (C-WS, T-OI, and T137-WS vs C-OI; C-WS+OI, T137-OI+OI, and T137-WS+OI vs C-OI+OI), the highest number of DEGs was identified in the comparisons C-WS vs C-OI and T137-WS+OI vs C-OI+OI, being 9,207 CDS upregulated and 12,118 downregulated, and 760 upregulated and 753 downregulated, respectively (**Supplementary Figure S3A**). However, the two comparisons with the lowest amounts of DEGs were T137-OI vs C-OI and T137-OI+OI vs C-OI+OI, with 104 CDS upregulated and 63 downregulated, and nine upregulated and one downregulated, respectively (**Supplementary Figure S3B**). Unique and shared DEGs obtained by comparisons of samples for WS and for recovery experiments are shown in Venn diagrams (**Figures 4A, B**). The largest number of unique DEGs, 12,217, was detected in the comparison C-WS vs C-OI, whereas 64 DEGs were shared among the three comparisons (**Figure 4A**). However, in the recovery experiment, T137-WS+OI vs C-OI+IO presented the largest number of unique DEGs, and just one (TraesCS3A03G0824100) was shared by the three comparisons (**Figure 4B**). These results indicate that WS was the primary factor driving the variation observed in the WS assay, whereas T137 played a key role in the recovery experiment. Although DEGs were found in all chromosomes and located on the three sub-genomes (**Supplementary Figure S4**), the highest number of DEGs for C-WS vs C-OI and T137-WS vs C-OI comparisons were on 2B and 2D, and 2D, respectively; whereas the lowest number was on 6A.

After DEGs functional annotation (**Figures 4C, D**), “carbohydrate metabolism”, “folding, sorting and degradation”, “protein families: genetic information processing”, and “signal transduction” were the KEGG functions most changed among comparisons. We observed a higher proportion of DEGs downregulated than upregulated related to “energy metabolism”, “signal transduction”, and “biosynthesis of secondary metabolites” KEGG categories, whereas there was a higher proportion of DEGs upregulated than downregulated for “genetic information processing” or “folding, sorting and degradation” in the comparisons C-WS vs C-OI and T137-WS vs C-OI (**Figure 4C**). These changes appear to be a consequence of the lack of irrigation suffered by the WS plants. However, in the comparison between T137-OI and C-OI, a distinct functional pattern was observed. Functional categories such as “carbohydrate metabolism”, “signal transduction”, “environmental adaptation”, and “protein families: signaling and cellular processes” showed a high proportion of upregulated DEGs. In contrast, “unclassified metabolism”, “amino acid metabolism”, and “protein families: metabolism” were predominantly associated with downregulated DEGs. Results show an impact of T137 application in the transcriptome of well-irrigated wheat plants. Moreover, “translation” and “protein families: genetic information processing” KEGG categories were highly represented within the downregulated DEGs in the comparisons of recovery treatments C-WS+OI or T137-WS+OI vs C-OI+OI (**Figure 4D**). CDS annotation was also performed for some GO terms associated with plant responses to abiotic stresses (**Supplementary Figure S5**). Although the proportion of GO terms investigated was similar between the comparisons C-WS vs C-OI and T137-WS vs C-OI, the number of DEGs for each GO was lower in the second one (**Supplementary Figures S5A, B**). In both comparisons, the response to ABA was the most represented GO, whereas the response to JA was the least (**Supplementary Figure S5A**). In addition, we found a higher proportion of DEGs related to “water transport” and “response to SA” GOs in the comparison T137-WS+OI vs C-OI+OI with respect to C-WS+OI vs C-OI+OI (**Supplementary Figure S5B**).

### *T. simmonsii* T137 modifies the wheat transcriptome in response to water stress and recovery upon rehydration

#### Photosynthesis-related DEGs and carbohydrate metabolism

**Figure 5:**
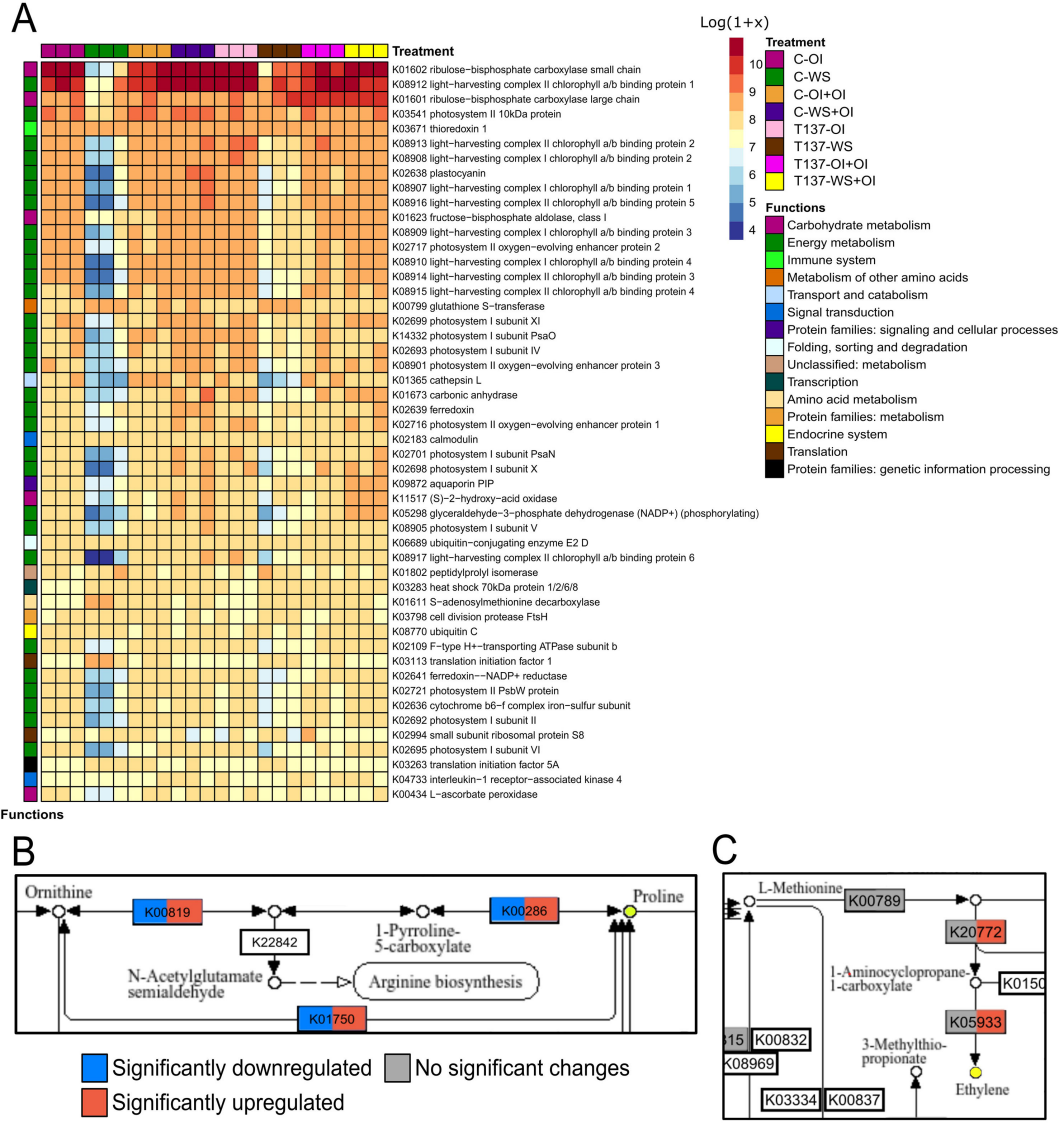
Main biological pathways involved in wheat plant responses to water stress (WS) mediated by *Trichoderma simmonsii* T137 after Kyoto Encyclopedia of Genes and Genomes (KEGG) enrichment. Heatmap of most expressed KEGGs overall, with their specific function, and expression values represented as the Log[Transcripts Per Kilobase Million (TPM) + 1] **(A)**. KEGG pathways of proline and methionine metabolism **(B, C)**. Colours represent differential regulation of DEGs involved in each process, being dark blue significantly downregulated, dark red significantly upregulated and grey without significant changes; with *|*log_2_ FoldChange*|* > 1 and *p* adjusted < 0.05. Within each KEGG box, colour on the left side represents T137-WS vs C-WS comparison and colour on the right side T137-WS vs T137-OI comparison.

Photosynthesis-related proteins are directly involved in drought responses, as their suppression at the onset of water deficit leads to the ROS generation, thereby inducing oxidative stress. As expected, WS caused an overall reduction in expression of photosynthesis-related genes in either T137-inoculated or untreated plants in comparison to C-OI plants (**Figure 5A**). More upregulated DEGs attributed to KEGG term “photosynthesis” and other related terms were detected in plants of the comparison T137-WS vs C-OI than in those of C-WS vs C-OI. Indeed, T137-WS plants showed upregulation of genes encoding light-harvesting complex II chlorophyll a/b binding protein 1 (LHCB1), phosphoribulokinase and photosystem II 10 kDa protein.

Under the OI condition, the “carbohydrate metabolism” KEGG category was overrepresented within upregulated DEGs in T137-OI plants with respect to C-OI (**Figure 4C**). Among the 16 DEGs, 15 were upregulated and a single alcohol dehydrogenase (TraesCS3A03G0077700) downregulated in T137-OI plants, suggesting that T137 is promoting carbohydrate metabolism in well-watered wheat plants. Nevertheless, minor differences were observed between C-WS and T137-WS plants, compared to C-OI, regarding the metabolism of carbohydrates. In addition, Rubisco genes were upregulated in *Trichoderma*-inoculated plants in comparison with those uninoculated, independently of the irrigation condition (**Figure 5A**), and all DEGs encoding the Rubisco small chain, a total of 25 genes, being upregulated in T137-WS plants when compared to C-WS.

#### Drought-related DEGs

WS increased the expression of well-known drought-induced genes, including those encoding the plant antioxidant machinery, aquaporins (AQP), LEA proteins, and osmolyte biosynthesis, among others (**Table 2**, **Supplementary Table S2**). The GO term “response to water deprivation (GO:0009414)” resulted in higher number of DEGs in C-WS than in T137-WS plants, when compared to C-OI plants, with 66 and 45 DEGs, respectively (**Supplementary Figure S5A**). Furthermore, the comparison T137-WS vs C-WS gave 27 DEGs related to “response to water deprivation”.

**Table 2:** Summary of wheat genes with their symbol and ID (version 2.1), and putative biological function, related to plant responses to water stress (WS), found differentially expressed between treatments [control (C)-optimal irrigation (OI), *Trichoderma simmonsii* T137 (T137)-OI, C-WS, T137-WS] from the WS experiment.

Function**	Gene symbol	Gene ID	T137-OI vs C-OI	C-WS vs C-OI	T137-WS vs C-OI	T137-WS vs C-WS
Antioxidants	*SOD2*	TraesCS4A03G0974600	-0.26	-2.52 (*)	-1.06	1.46
	*CAT*	TraesCS6A03G0093800	1.35 (*)	1.97 (*)	2.68 (*)	0.70
	*POX*	TraesCS7A03G1097700	-0.53	-11.91 (*)	-2.75 (*)	9.15 (*)
	*APX*	TraesCS2A03G1039900	0.33	-3.55 (*)	-1.74 (*)	1.80 (*)
PHP	*PAL*	TraesCS2B03G0540500	4.40	3.84	6.05 (*)	2.25 (*)
	*FLS*	TraesCS3A03G0837700	NA	6.27	8.01 (*)	1.71
AQP	*AQP-PIP*	TraesCS5A03G0802100	0.10	-5.17 (*)	-1.46 (*)	3.69 (*)
	*AQP-TIP*	TraesCS3D03G1192600	-0.03	-5.88 (*)	-2.85 (*)	3.01 (*)
Drought-related	*HSP*	TraesCS4A03G0203800	-0.31	8.63 (*)	6.43 (*)	-2.23 (*)
	*LEA*	TraesCS2B03G1193200	-0.69	8.25 (*)	4.51 (*)	-3.77 (*)
	*DHN*	TraesCS6D03G0772500	-0.58	9.16 (*)	6.60 (*)	-2.59 (*)
PA	*P5CS*	TraesCS3D03G0793300	-0.22	8.51 (*)	7.75 (*)	-0.79
	*P5CR*	TraesCS3B03G1339500	-0.07	4.58 (*)	3.10 (*)	-1.5
	*arg*	TraesCS2B03G0102100	-0.06	2.37 (*)	1.15 (*)	-1.24 (*)
	*OAT*	TraesCS5A03G0900400	-0.27	4.22 (*)	2.12 (*)	-2.12 (*)
	*ODC1*	TraesCS5A03G0811400	NA	7.99 (*)	6.87 (*)	-1.13
	*speE*	TraesCS7B03G0654200	-0.07	1.19 (*)	1.99 (*)	0.78
ABA	*NCED*	TraesCS5B03G0943700	0.14	5.86 (*)	5.12 (*)	-0.76
	*ABA2*	TraesCS2A03G0254300	-0.21	6.87 (*)	5.37 (*)	-1.53 (*)
ET	*ACS*	TraesCS2D03G0897400	0.49	3.89 (*)	4.80 (*)	0.89
	*EIN2*	TraesCS4D03G0060200	0.66	0.03	1.36 (*)	1.31 (*)
JA	*LOX*	TraesCS5B03G0014800	-0.06	-2.13	3.02 (*)	5.14 (*)
	*AOS*	TraesCS4B03G0647300	-1.11	2.19	5.47 (*)	3.24 (*)
	*AOC*	TraesCS6B03G1030800	-0.70	-0.22	1.13 (*)	1.33 (*)
	*JAZ*	TraesCS7A03G0474000	NA	5.08 (*)	7.85 (*)	2.58 (*)
GA	*GA20ox*	TraesCS5B03G1356500	1.03	-8.13 (*)	-1.27	6.85 (*)
	*GID1*	TraesCS1D03G0617800	0.32	0.85 (*)	3.52 (*)	2.65 (*)
IAA	*YUCCA*	TraesCS5B03G1370600	-0.36	-8.88 (*)	-1.16	7.71
	*TAA1*	TraesCS3D03G0184300	-0.21	-9.82 (*)	-1.48 (*)	8.33 (*)
	*IAA*	TraesCS5B03G0146300	0.39	-3.70 (*)	-0.70	3.00 (*)
	*SAUR*	TraesCS7D03G0167400	1.35	-7.91 (*)	-1.88	5.91 (*)
SL	*DWARF27*	TraesCS7D03G0971000	-0.18	-8.64 (*)	-1.22	7.40 (*)
	*CCD*	TraesCS5D03G0005200	0.48	-8.33 (*)	-2.02	6.30
	*CYP711A1*	TraesCS6A03G0470400	0.04	-4.70 (*)	-3.43 (*)	1.29
CK	*CRE1*	TraesCS4D03G0091900	0.13	-2.71 (*)	-0.88 (*)	1.81 (*)
BR	*DET2*	TraesCS3A03G0869900	0.14	-8.60 (*)	-1.98 (*)	6.61 (*)
	*CYP92A6*	TraesCS5B03G0168300	0.30	-8.90 (*)	-0.62	8.26 (*)
	*BRI1*	TraesCS7A03G1324900	0.10	-8.49 (*)	-2.61 (*)	5.99 (*)
Signal transduction	*CDPK*	TraesCS2A03G0416900	0.42	0.16	1.41 (*)	1.23 (*)
	*MAPK*	TraesCS7A03G0826400	-0.32	-2.73 (*)	1.12	3.83 (*)
	*PP2C*	TraesCS1D03G0967400	NA	7.36 (*)	4.65 (*)	-2.77 (*)
	*LRR-RLK*	TraesCS7A03G0388200	-0.13	-8.56 (*)	-1.07	7.48 (*)

** PHP, phenylpropanoid; AQP, aquaporin; PA, polyamine; ABA, abscisic acid; ET, ethylene; JA, jasmonic acid; GA, gibberellin; IAA, indole-acetic acid; SL, strigolactone; CK, cytokinin; BR, brasinosteroid; NA, not available.

Values correspond to log_2_ FoldChange values and significance is indicated by * (*p* adjusted value < 0.05).

WS caused a global upregulation of genes encoding antioxidant enzymes, including *SOD*, glutathione peroxidase (*GSH*), *CAT* and some class III *POX* (**Table 2**, **Supplementary Table S2**), and it was reverted after rehydration. However, most *POX* genes were highly upregulated in the comparison T137-WS vs C-WS (**Table 2**, **Supplementary Table S2**). Several genes encoding ascorbate peroxidases (*APX*) were downregulated in C-WS but not in T137-WS plants with respect to C-OI plants. WS but also T137 inoculation increased the expression of phenylalanine ammonia lyase (*PAL*) genes. However, WS reduced flavanone biosynthesis genes, as seen in the decreased expression of chalcone synthase (*CHS*) and chalcone isomerase (*CHI*) genes. In addition, *CHS* and *CHI* genes were downregulated to a greater extent in C-WS than T137-WS plants. Among DEGs encoding flavonol synthase (*FLS*), 40% were upregulated in the comparison C-WS vs C-OI, while 60% were upregulated in the comparison T137-WS vs C-OI. Nevertheless, anthocyanins biosynthesis seems to be promoted to a larger extent in C-WS than T137-WS plants, as observed from expression of dihydroflavonol 4-reductase (*DFR*) and anthocyanidin synthase (*ANS*) genes.

WS broadly enhanced the expression of genes related to osmolyte biosynthesis, including proline, glycine betaine, polyamines, and osmotically active carbohydrates, such as trehalose. Proline biosynthesis genes were upregulated by WS in both C-WS and T137-WS treatments compared to C-OI. However, C-WS plants showed higher expression in delta-1-pyrroline-5-carboxylate synthetase (*P5CS*), *P5CR* and ornithine aminotransferase (*OAT*) genes than T137-WS plants (**Figure 5B**). The glycine betaine biosynthesis *BADH* gene was upregulated by WS, but no differences were observed between C-WS and T137-WS treatments; whereas a choline monooxygenase (*CMO*) gene was downregulated by WS in C-WS, but not in T137-WS plants when compared to C-OI. Overall, polyamine biosynthesis gene expression was promoted by WS to a greater extent in C-WS than in T137-WS plants. WS also increased the expression of trehalose biosynthesis genes, including trehalose 6-phosphate synthase (*TPS*) and trehalose 6-phosphate phosphatase (*TPP*). Remarkably, a *TPP* gene (TraesCS5A03G0508000) was upregulated in the comparison T137-WS vs C-WS.

Under the WS condition, 79% of DEGs encoding AQPs were downregulated in the C-WS treatment, whereas 71% were downregulated in T137-WS when compared to C-OI. All genes encoding nodulin-26 like intrinsic proteins (*NIPs*) and small basic intrinsic proteins (*SIPs*) were downregulated by WS with minor differences between C-WS and T137-WS plants. Genes encoding plasma membrane intrinsic proteins (*PIPs*) were downregulated by WS to a greater extent in C-WS than in T137-WS plants after comparing to C-OI (**Table 2** and **Supplementary Table S2**). However, in turn, all *PIP* genes were upregulated in the comparison T137-WS vs C-WS. Expression of genes encoding tonoplast intrinsic proteins (*TIPs*) was generally increased by WS, with minor differences between C-WS and T137-WS plants. Likewise, in the comparison T137-WS vs C-WS, seven of the nine DEGs related to the “water transport” GO term (GO: 0006833) were upregulated, while only two were downregulated, indicating an increase of AQP activity in T137-WS plants (**Supplementary Figure S5A**). Regarding DEGs encoding heat shock proteins (HSP), in comparison with the C-OI treatment, all DEGs were upregulated in the T137-WS plants, but only 70% were upregulated in C-WS (data not shown). Additionally, gene sequences encoding LEA proteins and dehydrins annotated in the wheat genome (IWGCS v2.1) were retrieved from the Phytozome database (accessed on April 18, 2025) and manually searched in our dataset. As a result, most DEGs encoding LEA proteins and dehydrins were upregulated by WS to a greater extent in C-WS than in T137-WS plants (**Supplementary Table S2**).

#### Phytohormone-related DEGs

We have observed that expression of several phytohormone pathway genes was affected by the application of WS or T137, including ABA, SA, ET, JA, IAA, GAs, SLs, CKs and BRs (**Table 2** and **Supplementary Table S2**, **Figure 5C**). ABA biosynthesis seems to be enhanced by WS in wheat plants, as inferred from the upregulation of 9-cis-epoxycarotenoid dioxygenase (*NCED*), xanthoxin dehydrogenase (*ABA2*) and aldehyde oxidase 3 (*AAO*) genes. Remarkably, no expression differences were found in either ABA biosynthesis or ABA receptor genes in the comparison T137-WS vs C-WS. In addition, all genes related to the GO term “response to ABA” (GO:0009737) were downregulated in T137-WS plants with respect to C-WS (**Supplementary Figure S5A**). Likewise, WS increased the expression of ET biosynthesis genes, such as those encoding ACC synthase (ACS) and ACC oxidase (ACO), and no differences were observed between C-WS and T137-WS treatments.

Under the OI condition, T137 application altered SA-related gene expression (**Supplementary Figure S5A**). WS caused an overall expression increase of SA biosynthesis genes in both C-WS and T137-WS treatments compared to C-OI, as observed for PAL and isochorismate synthase genes, although the highest expression levels of PAL genes were detected for T137-WS (**Table 2** and **Supplementary Table S2**). WS also increased the overall expression of JA biosynthesis genes, compared to C-OI plants. Additionally, T137-WS plants showed higher expression levels than C-WS for JA biosynthesis genes, including those encoding LOX, allene oxide synthase (AOS) and allene oxide cyclase (AOC). WS drastically inhibited the expression of GA biosynthesis genes in C-WS plants, including those encoding GA20 oxidase (GA20ox) and GA13ox, but this effect was mitigated in T137-WS plants, when compared to C-OI. In this study, WS reduced the expression levels in IAA biosynthesis genes to a greater extent in C-WS than in T137-WS plants, compared to C-OI plants. Particularly, genes encoding indole-3-acetamide hydrolase (*AMI1*), flavin monooxygenase (*YUC*) and tryptophan aminotransferase (*TAA1*) were strongly downregulated by the WS. Interestingly, a *TAA1* gene (TraesCS3D03G0184300) was highly upregulated in the T137-WS vs C-WS comparison.

Regarding SL pathways (**Table 2**, **Supplementary Table S2**), WS caused an overall reduction in the expression of biosynthesis genes, such as those encoding all-trans-/9-cis-β-carotene isomerase (*DWARF27*), carotenoid cleavage dioxygenases (*CCD*) and cytochrome P450 CYP711A, compared to C-OI plants. Similar behaviour was observed in BR biosynthesis genes, since WS decreased the expression of those encoding cytochrome P450 CYP90A1, steroid 5-alpha-reductase (*DET2*), typhasterol/6-deoxotyphasterol 2alpha-hydroxylase (*CYP92A6*) and brassinosteroid 6-oxygenase (*CYP85A1*). Regarding CK biosynthesis, the cytokinin trans-hydroxylase *CYP735A* (TraesCS7B03G0638400) gene expression was reduced to a greater extent in C-WS than in T137-WS plants, compared to C-OI.

#### Rehydration-related DEGs

Rehydration had more impact on the transcriptome of C-WS+OI and T137-WS+OI plants, than in that of unstressed T137-OI+OI plants, compared to C-OI+OI. When compared to C-OI+OI (**Supplementary Table S3**), T137-WS+OI plants presented more DEGs than C-WS+OI, with 1,513 vs 1,256. Particularly, 90% of DEGs obtained from the T137-WS+OI vs C-WS+OI comparison was found to be upregulated, including genes encoding beta-glucosidase, alpha-1,4-galacturonosyltransferase, ribulose-bisphosphate carboxylase large chain, alpha-amylase, inositol 3-alpha-galactosyltransferase, and raffinose synthase. In addition, genes encoding the photosystem II PsbH protein were upregulated by T137 in rehydrated plants, compared to untreated plants. Most DEGs related to “lipid metabolism” (six out of eight DEGs) were upregulated in T137-WS+OI plants compared to C-WS+OI. Furthermore, T137 application increased expression in rehydrated plants of two DEGs related to “replication and repair” (DNA mismatch repair protein MSH6 and ATP-dependent DNA helicase 2 subunit 1). Concerning DEGs encoding POX enzymes obtained from T137-WS+OI vs C-WS+OI comparison, two were upregulated and one downregulated. All DEGs encoding AQPs were upregulated in T137-WS+OI plants, while only one DEG was upregulated in C-WS+OI plants, compared to C-OI+OI. Among the DEGs obtained from T137-OI+OI vs C-OI+OI comparison, several genes encoding Rubisco large chain, acid phosphatase type 7, interleukin-1 receptor-associated kinase 1, adenosylhomocysteine nucleosidase, and ATP-dependent metalloprotease were upregulated. DEGs related to carbohydrate metabolism were upregulated to a greater extent in T137-WS+OI than in C-WS+OI plants compared to C-OI+OI (**Figure 5A**).

#### Differentially transcribed transcription factors

**Figure 6:**
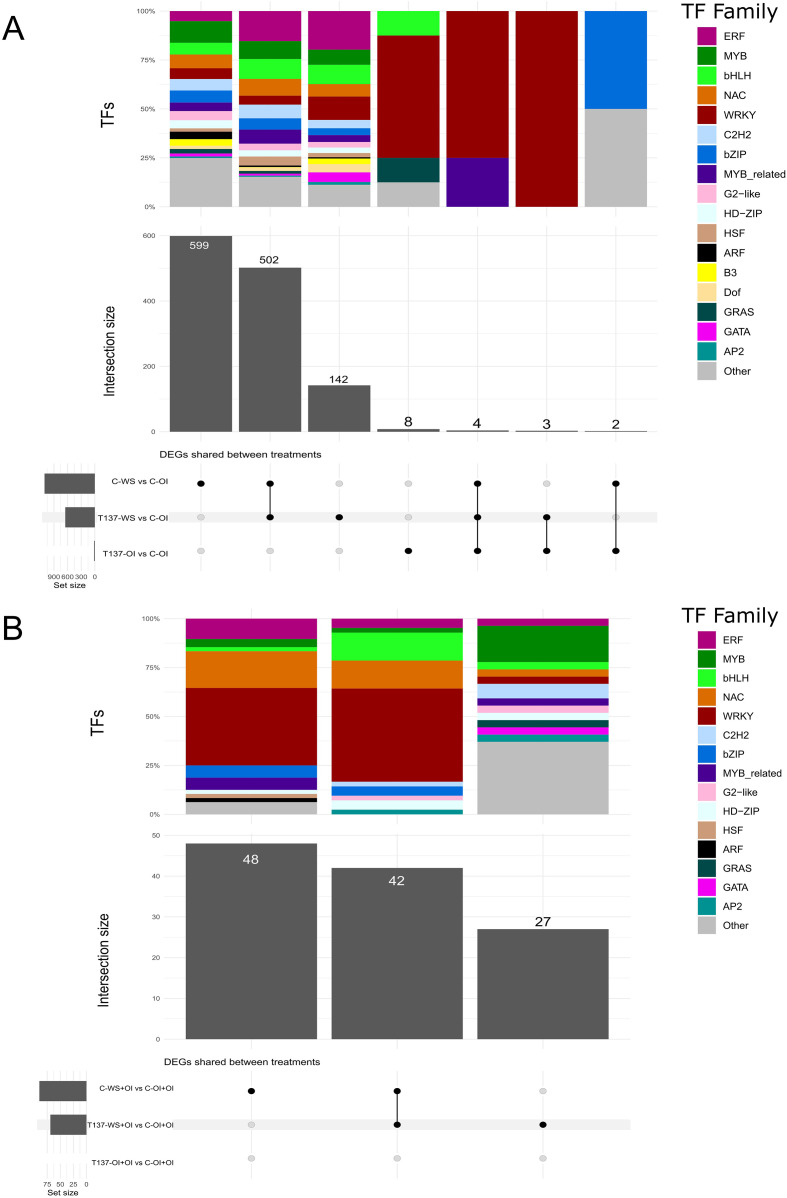
Upset plot showing the number of shared and unique differentially expressed genes encoding transcription factors (TFs) between comparisons and their TF family proportion. Water stress experiment **(A)**. Recovery experiment **(B)**.

To gain deeper insight into the molecular pathways underlying plant responses to drought and the changes caused by the application of strain T137, an analysis of TFs was conducted. After comparing with their respective controls, C-OI and C-OI+OI, 1, 260 and 117 TFs were found within the DEGs identified in the WS and recovery experiments, respectively. Overall, a high representation of MYB, bHLH, NAC, WRKY and C2H2 gene families was observed in C-WS and T137-WS plants with respect to C-OI (**Figure 6A**), and genes encoding NAC, WRKY, bZIP and MYB-related factors were the most abundant in C-WS+OI and T137-WS+OI plants compared to C-OI+OI (**Figure 6B**).

**Figure 7:**
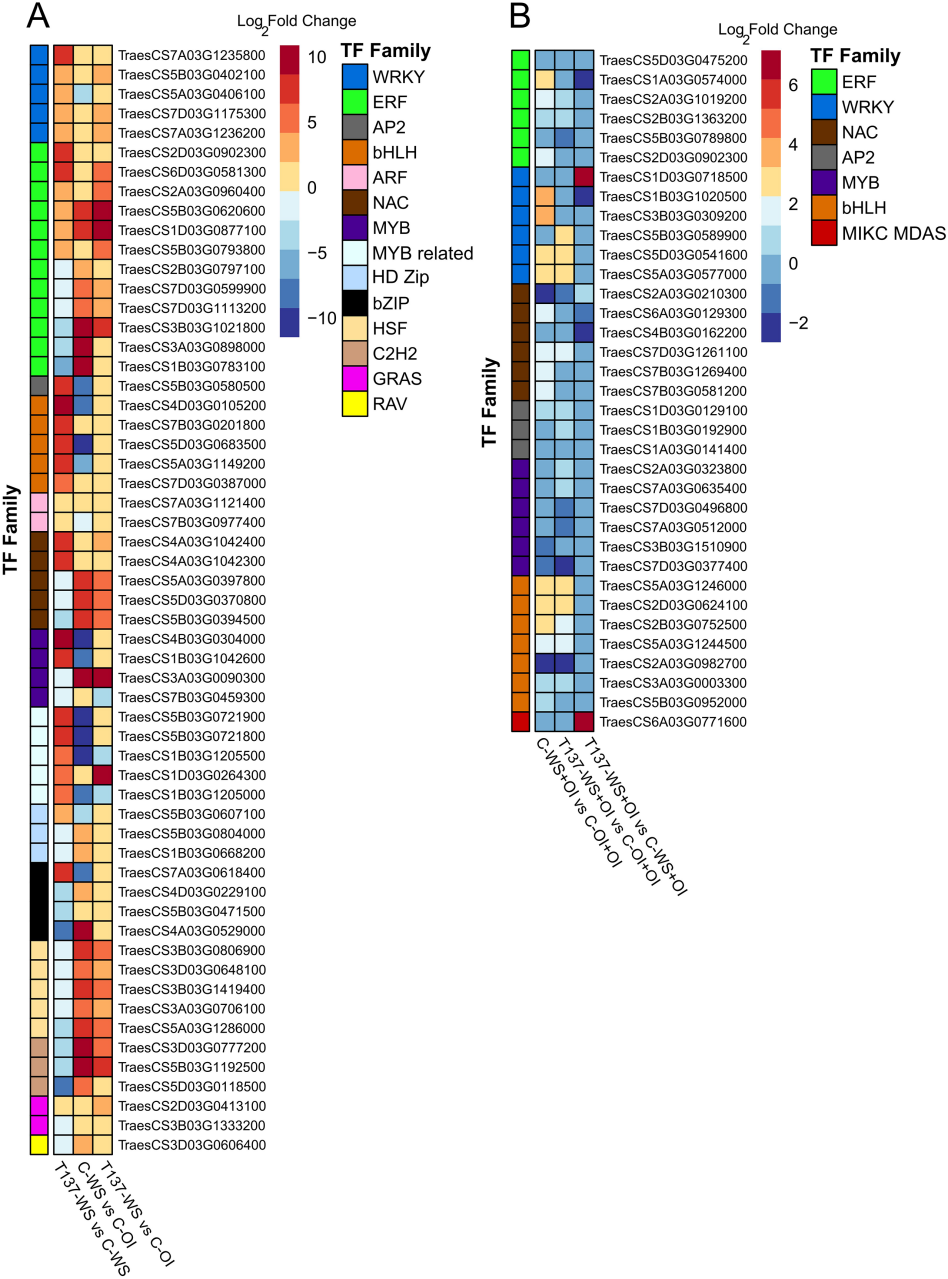
Heatmap of differentially expressed genes encoding transcription factors previously annotated in wheat. Water stress experiment **(A)**. Recovery experiment **(B)**. Log_2_ FoldChange values from each comparison are used to plot the heatmap, with *|*log_2_ FoldChange*|* > 1 and *p* adjusted < 0.05. The dark red colour shows the highest values and the dark blue colour indicates the lowest ones.

Under the WS condition and compared to C-WS plants, genes encoding members of WRKY, bHLH, AP2, auxin response factor (ARF) and MYB-related TF families were upregulated in T137-WS plants, while genes encoding NAC, HSF, HD-ZIP, C2H2 and bZIP factors were downregulated (**Figure 7A**). Under the rehydration condition, DEGs encoding WRKY and MIKC-type MADS-box members were upregulated in T137-WS+OI plants, while those encoding ethylene response factor (ERF) and NAC were downregulated, compared to C-OI+OI plants (**Figure 7B**). Under the OI condition, 17 TFs were differentially regulated as a consequence of T137 application. Indeed, all DEGs encoding WRKY (11 genes in total) were upregulated in the comparison T137-OI vs C-OI. Particularly, eight genes related to WRKY, bHLH and GRAS factor families were exclusively differentially expressed in the comparison T137-OI vs C-OI (**Figure 6A**). Notably, no TF genes were differentially expressed in the comparison T137-OI+OI vs C-OI+OI (**Figure 6B**).

**Figure 8:**
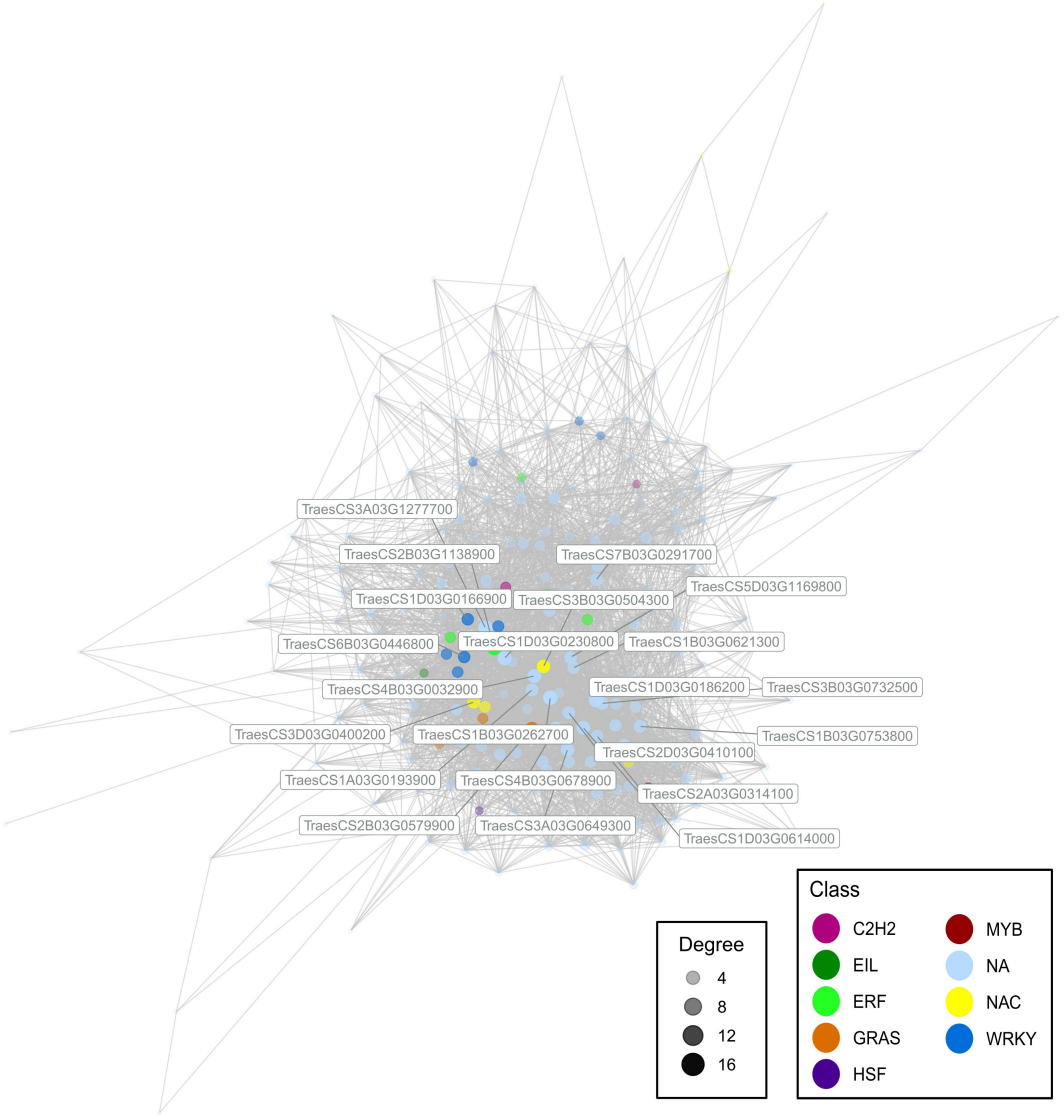
Module containing high proportion of environmental adaptation genes, which was extracted from a weighted gene co-expression network analysis using Transcripts Per Kilobase Million data from the 24 cDNA library samples. Genes were identified as hub by their module membership and degree, and coloured according to the related transcription factor family. Only genes with a correlation greater than 0.8 are visualised.

A WGCNA was performed to further understand the relationship between gene transcriptional profiles. We obtained 30 modules, ranging from 36 genes the smallest to 5,382 the largest. We focused on a module rich in genes linked with environmental adaptation and positively correlated with the T137-WS treatment (0.45*), which was composed of 219 genes (**Figure 8**). In this module, we found hub genes encoding TF family members, such as WRKY (TraesCS1D03G0166900, and TraesCS6B03G0446800), NAC (TraesCS3B03G0504300, and TraesCS3D03G0400200), ERF (TraesCS2B03G1138900), and GRAS (TraesCS2B03G0579900) as well as other genes encoding factors for plant responses to stress.

### RNA-Seq and qPCR data were positively correlated

**Figure 9:**
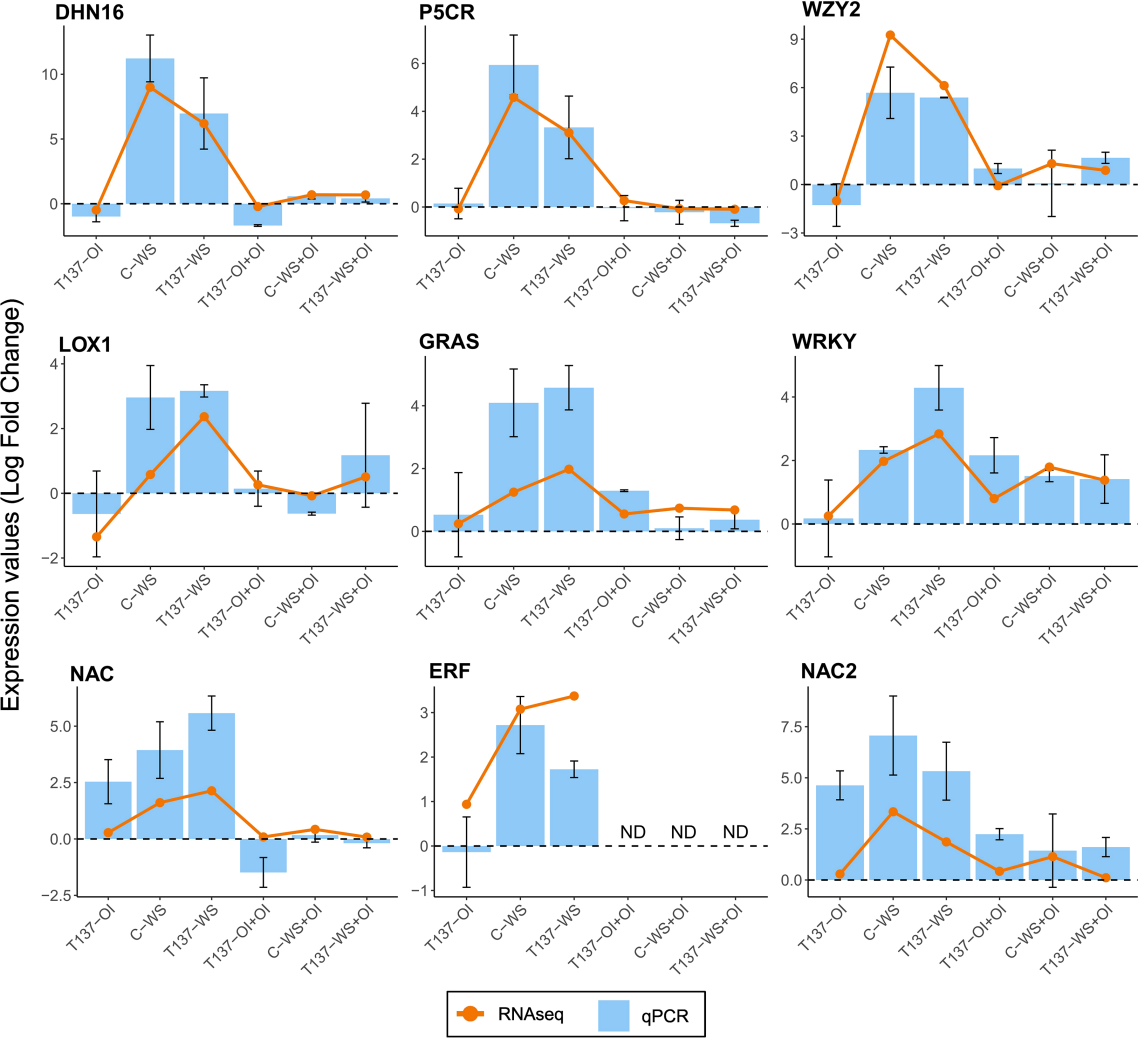
Correlation between RNA-sequencing (RNA-Seq) and qPCR data for eight wheat genes, indicating of the putative protein they encode. Bars and lines show log_2_ FoldChange values of qPCR data and RNA-Seq data for each gene, respectively. The bars represent mean values ± standard deviation of three independent biological replicates.

The RNA-Seq results were validated through qPCR using eight genes related with plant response to WS. All of them were upregulated in the samples from C-WS or T137-WS plants compared to C-OI, and no substantial changes were detected in rehydrated plant samples (**Figure 9**). After comparison of log_2_ FoldChanges measures from RNA-Seq analysis with relative expression levels from qPCR analysis, expression trends agreed and were in concordance (R^2^ = 0.73).

## Discussion

Drought is a relevant abiotic stress that often limits plant growth and has a strong impact on crop yields. Limited water availability in the soil has been related to the induction of oxidative and osmotic stresses that negatively affect plant growth (Sharma et al., 2019; Bandurska, 2022), although beneficial microorganisms can help to mitigate these constraints (Liu et al., 2020; Illescas et al., 2022; Aljeddani et al., 2024). As we previously reported, *T. simmonsii* T137 can attenuate WS negative effects in wheat plants (Pedrero-Méndez et al., 2021), and we are now confirming that the application of this strain exerts protection against WS. In the present study, both T137 and *T. asperellum* T25 have shown their ability to increase RWC and reduce oxidative stress by activating the antioxidant enzyme machinery in WS wheat plants. The higher aboveground FW and DW values with respect to control, only detected for T137, indicate that growth was less affected by WS in plants treated with T137 than with T25. These results with T137 agree with those observed in cocoa, rice, tomato and wheat plants, in which the *Trichoderma* application, caused a delay in the decline of growth-related parameters of plants subjected to WS (Bae et al., 2009; Shukla et al., 2012; Khoshmanzar et al., 2020; Aljeddani et al., 2024).

Plants accumulate osmolytes and non-enzymatic antioxidants to counteract the negative effects of WS (Sharma et al., 2019; Bandurska, 2022). A significant increase of proline accompanied by increased MDA levels, a marker of membrane lipid peroxidation damage, was detected in C-WS, T25-WS and T137-WS plants. However, such an increase was lower in plants pretreated with *Trichoderma*. Similar observations for proline and MDA accumulation have been described in rice leaves of plants treated with *Trichoderma* (Shukla et al., 2012), and in tomato plants only for MDA (De Palma et al., 2021). These findings, together with reduced H_2_O_2_ levels, increased SOD, POX and CAT activities, and a healthier phenotype observed in WS *Trichoderma*-treated plants in comparison to those of the WS control, are indicative of a protective effect of both *Trichoderma* strains on wheat plants against drought, which are shown to be subjected to less oxidative stress. A stimulation of antioxidant enzymatic activities by the use of *T. harzianum* in combination with biogenic silica nanoparticles has been also associated with enhanced wheat productivity under drought (Aljeddani et al., 2024). We have also observed that the lipid peroxidation damage generated in plants during the WS period was not reversed by rehydration. However, T25-WS+OI and T137-WS+OI plants showed lower MDA content than those of the control (C-WS+OI), as well as T137-WS+OI plants had higher SOD activity than the control. These results indicate that strain T137 has a positive effect on the recovery of wheat plants upon rehydration.

The protective effect of *Trichoderma* against abiotic stresses like drought and salinity has been already reported in different plants (Bae et al., 2009; Mastouri et al., 2012; Brotman et al., 2013; Rubio et al., 2014), including wheat (Zhang et al., 2019; Pedrero-Méndez et al., 2021; Illescas et al., 2022; Aljeddani et al., 2024). Here, we have studied the whole transcriptomic differences of T137-treated and untreated winter bread wheat plants when facing WS, and in recovery after rehydration.

When the samples from the WS and recovery experiments were analysed jointly and separately, tSNE clustering showed that WS is the main driver of transcriptional changes detected in the wheat plants. However, T137-WS+OI samples clustered closer to those unstressed than those of C-WS+OI, suggesting that *Trichoderma* minimizes damage derived from the lack of irrigation. Conversely, in the recovery experiment, we detected the highest number of DEGs for the comparison T137-WS+OI vs C-OI+OI whereas no differences in phenotype were observed among plants from the different treatments. The decline observed in the number of DEGs, and particularly in those encoding TF family members, in T137-WS vs C-OI with respect to C-WS vs C-OI is indicative of an extensive downregulation in the transcriptome of WS wheat plants caused by T137 application. This agrees with physiological and biochemical data recorded under the WS condition, as has been previously described in *T. longibrachiatum*-tomato responses to drought (De Palma et al., 2021).

As expected for a severe WS condition, photosynthetic assimilation seems to be decreased regardless of whether plants were pretreated with *Trichoderma* or not. Nevertheless, the KEGG enrichment showed upregulation of genes for “photosynthesis” and other related terms in T137-WS with respect to C-WS plants, which would agree with the observed phenotypes and calculated growth data from the WS experiment. This fact is reinforced by the metabolic functions related to carbohydrate metabolism [e.g. Rubisco (K01601, K01602) and fructose-bisphosphate aldolase (K01623)], photosystem complexes [e.g. LHCB1 (K08912), LHCB2 (K08913), psbR (K03541)] and immune system [e.g. thioredoxin trxA (K03671)], which accounted most of the overrepresented KEGGs in T137-WS plants, but having a recurrent pattern of downregulation in C-WS plants. *Trichoderma* application also enhanced carbohydrate metabolism in well-irrigated plants, with upregulation of Rubisco activity independently of the irrigation condition, as previously described in tomato plants (Geng et al., 2022).

As previously reported (Sharma et al., 2019; Bandurska, 2022), wheat plants responded to WS by activating their enzymatic system to avoid ROS accumulation toxicity. Here, regardless of T137 application, a high number of genes encoding redox enzymes such as GSH (gluthatione peroxidase), SOD, CAT and APX were significantly upregulated in plants subjected to WS. In contrast to the biochemical activity measures, the RNA-Seq analysis did not detect significant changes for *SOD* and *CAT* genes between T137-WS and C-WS plants, and only an upregulated *APX* (TraesCS2A03G1039900) gene was detected in T137-WS. Such a discrepancy could be a consequence of data generated by different methodologies, and because the transcription and translation times are obviously different. Class III POX can display multifaceted roles in plants, since in addition to scavenging ROS, they are involved in lignin and suberin formation, and cell wall elongation (Passardi et al., 2004). Interestingly, a group of *POX* genes, which was strongly downregulated by WS, presented significantly less downregulation in T137-WS plants. This again would agree with the lower growth limitation, in terms of FW, observed in T137-treated plants in response to WS. Plants growing under stressful environments activate phenylpropanoid pathway resulting in accumulation of phenolic compounds with potential to scavenge ROS (Sharma et al., 2019). *PAL* genes are implicated in this pathway, leading to the formation of precursors of several metabolites such as flavonoids, anthocyanins, and SA, which act as key signalling molecules involved in plant development and defence responses (Dixon et al., 2002). We detected in WS T137-treated plants, four PAL and two flavonol biosynthesis genes upregulated, but not the activation of SA-dependent defence marker genes. These results could support the fact that phenylpropanoid pathway activated by *Trichoderma* may be contributing to the reduced ROS levels observed in T137-WS plants, and this is in accordance with what has been described for *T. harzianum*-treated wheat plants infected by *Bipolaris sorokiniana* (Singh et al., 2019). However, it has also been described that heat shock factors promote accumulation of flavonoids, ROS scavenging, and plant survival under drought conditions (Wang et al., 2020), and even HSFB1 coordinates plant growth and drought tolerance (Zheng et al., 2025). As we have detected downregulation of numerous *HSF* and *HSP* genes and other encoding ABA-related proteins in the comparison T137-WS vs C-WS, flavonoid and ABA accumulation does not seem to occur in T137-WS plants, since they are in better physiological condition.

In agreement with the healthy phenotype of WS T137-treated plants, corroborated by biochemical measurements, we detected downregulation of a high number of genes associated with biosynthesis of osmolytes, such as proline and trehalose, and highly hydrophilic proteins, such as LEA and dehydrins, which are accumulated by plants to mitigate adverse effects of drought (Kim et al., 2005; Brini et al., 2007; Garg and Pandey, 2016; Chen et al., 2019), while polyamine metabolism was not modified. Similar observations for proline and polyamines have been reported in WS *Trichoderma*-treated rice, tomato and wheat plants (Shukla et al., 2012; De Palma et al., 2021), while upregulation of proline and trehalose biosynthesis genes has been described in mycorrhized wheat plants subjected to salt stress (Puccio et al., 2023).

We have also observed in T137-WS plants, two upregulated DEGs encoding calcium-dependent protein kinases (CDPKs), which have been reported to be involved in Ca^2+^ signal transduction in abiotic stress responses in rice, apple, or tomato plants (Xiang et al., 2007; Hu et al., 2016). We also detected that the application of T137 induced the upregulation of numerous *PIP* and *TIP* genes, which encode aquaporins known to play key roles in plant water-use efficiency and water transport under salt and water stress conditions (Aroca et al., 2007; Chen et al., 2017). Our observations on *TIP* genes coincide with that reported in salt-stressed mycorrhizal wheat plants (Puccio et al., 2023). Interestingly, a higher proportion of DEGs related to the GO term “water transport”, and upregulation of genes encoding PIP proteins, were detected in T137-WS+OI plants with respect to those of C-WS+OI, which seems to indicate that *Trichoderma* favours water availability by the plant in the recovery condition.

Although ABA is considered the main phytohormone in regulating plant processes to face the drought, others such as ET, SA, JA, auxins, GAs, CKs, and BRs are also important, and they usually all maintain a complex crosstalk network to increase the plant survival under drought conditions (Rubio et al., 2017; Ullah et al., 2018). CDPKs have functions in various aspects of plant growth and development, as well as biotic and abiotic stress responses, since they regulate GAs homeostasis, affect the auxin transport and responses to auxin signalling, respond to ET and affect its biosynthesis, regulate JA biosynthesis, and participate in the response to ABA signalling (Shi et al., 2018; Wei et al., 2025). In the comparison C-WS vs C-OI, one gene (TraesCS7A03G0620400) encoding CDPK, two encoding MAPK and three encoding phosphatase PP2C, all of them involved in signal transduction, were strongly upregulated. Additionally, several genes related to ABA biosynthesis, as well as several genes related to biosynthesis and signalling of ET, were also upregulated. In parallel, a high number of genes involved in GA, auxin, and SL biosynthetic pathways, as well as genes for both transport and response to auxin signalling were repressed, while two genes of JAZ, transcriptional repressors of JA signalling, were induced. These results indicate that C-WS plants prioritize defence vs growth and are consistent with what could be expected for plants responding to drought (Hirayama and Shinozaki, 2007; Singh and Roychoudhury, 2023). BRs play an essential role in acclimation to environmental stress, resulting in increased crop yield and plant growth (Kim and Russinova, 2020). In our study, we have seen in the comparison C-WS vs C-OI that numerous genes encoding cytochrome CYP, involved in BR biosynthesis, three genes encoding membrane receptor BRI1 and four genes encoding LRR receptor-like kinases (LRR-RLKs), which function as BRI1 co-receptors, were downregulated. This agrees with what was above indicated for GAs and auxins, and with the lowest FW value recorded for C-WS plants.

*Trichoderma* communicates with the plant in a way that contributes to phytohormone signalling networks, producing its own phytohormones and modifying their balance in the plant (Contreras-Cornejo et al., 2009; Viterbo et al., 2010; Jaroszuk-Ściseł et al., 2019; Illescas et al., 2021). Particularly, increased production of IAA and ABA, as well as ACC deaminase activity by *Trichoderma* have been correlated with enhanced tolerance to abiotic stress in wheat plants treated with the fungus (Zhang et al., 2019; Pedrero-Méndez et al., 2021; Rauf et al., 2021; Illescas et al., 2022). Pre-treatment with T137 highlighted the role of *Trichoderma* in modifying wheat plant responses to WS. As observed in the T137-WS vs C-WS comparison, the increased expression of numerous genes encoding proteins involved in biosynthesis and signalling of GAs, auxins, BRs, or SLs, such as LOX, AOS, AOC, GA20ox, GA13ox, GID1, AMI1, TAA1, YUCCA, DRARF27, CYP92A6, CDPK, BRI1, and LRR-RLK, together with the reduced expression of one gene coding for ABA2 and tree coding for phosphatases PPC2, and the resulting phenotype, confirm the minimal activation of drought stress response pathways in T137-WS plants.

Many genes encoding TFs belonging to 14 families, such as WRKY, ERF, NAC, bZIP, bHLH, or HSF, with an important role in plant responses to biotic and abiotic stresses (Singh et al., 2002; Nakashima et al., 2012; Kazan and Manners, 2013), were found to be differentially expressed in plants from pairwise comparisons between treatments of the WS experiment. The significant expression changes observed for 57 TF genes, including those encoding master regulators, between C-WS vs C-OI and T137-WS vs C-WS comparisons, are indicative of an important role of *Trichoderma* modifying the biological networks of WS wheat plants. Here, we have identified TF genes upregulated by the T137 application, such as five encoding WRKY, five bHLH, two NAC, five MYB-related, and one bZIP. Such upregulation has been described in salt-stressed mycorrhizal-wheat symbiosis (Puccio et al., 2023). The overexpression of genes coding for WRYK, NAC, or bZIP enhanced responses of rice, tobacco, and wheat plants to abiotic stresses (Hu et al., 2006; Shi et al., 2014; Xu et al., 2015; Bi et al., 2021). Particularly, within the bHLF family, we also identified, two genes encoding PIF3 and one (TraesCS5A03G1149200) encoding MYC2 that were upregulated. Moreover, a downregulation of six genes encoding ERF, which act as key regulatory hubs, integrating ET, ABA, JA, and redox signalling in the plant response to several abiotic stresses (Muller and Munne-Bosch, 2015), and of five encoding HSF, which are involved in flavonoid synthesis and ABA signalling (Wang et al., 2020), was also observed in T137-WS plants, indicating a repression of ET and ABA responses. MYC2 is a master switch regulator of positive and negative interplay between ABA and JA signaling (Liu and Avramova, 2016). In our study we have also seen that T137-WS plants had a behaviour compatible with the defence-growth model of *Trichoderma* interaction with plants (Hermosa et al., 2013), as they showed upregulation of genes related to GAs, auxins, and BRs, indicative of plant growth stimulation, while genes encoding JAZ repressor proteins were also upregulated. Sufficient levels of JAZs make them available to interact with MYC2 to suppress the transcription of JA-responsive genes involved in defence responses (Kazan and Manners, 2013), while repress the activity of DELLA proteins on the TF PIF3, so growth promotion is possible (Kazan and Manners, 2012). Then, the observed upregulation of JA biosynthesis pathway genes would generate an increase in JA levels that could trigger the destruction of such JAZ excess, resulting in the release of MYC2 and a balance of plant growth/defence responses.

Since RNA-Seq provides information on a large number of genes, especially in genomes as huge and complex as the wheat genome, we performed a WGCNA to understand the gene expression relationships and identify key regulator genes, known as hub genes. So, when the eight treatments were analysed and considered as a whole, two *WRKY*, two *NAC*, one *ERF* and one *GRAS* were identified as hub genes within the module including 219 genes related to environmental adaptation, aiming at the maintenance of phytohormonal homeostasis to balance the growth and defence responses of wheat plants.

Results contribute to understanding how wheat plants treated with *T. simmonsii* T137 sense and respond to water scarcity and how they recover upon rehydration, which makes *Trichoderma* relevant to optimize its application to rainfed wheat crops in arid areas.

## Data Availability

The datasets presented in this study can be found in online repositories. The names of the repository/repositories and accession number(s) can be found in the article/**Supplementary Material**.

## Supplementary material

Supplementary Table 1 Primer pairs used for quantitative real time PCR, with forward and reverse sequences and gene name with sources.

Supplementary Table 2 Wheat gene summary with their symbol and ID (version 2.1), and putative biological function, associated with plant responses to water stress (WS), found differentially expressed between treatments [control (C)-optimal irrigation (OI), *Trichoderma simmonsii* T137 (T137)-OI, C-WS, T137-WS] from the WS experiment. Values correspond to Log_2_ Fold change values and significance is indicated by * (*p* adjusted value < 0.05).

Supplementary Table 3 Compilation of wheat genes, including their symbol and corresponding ID (version 2.1), along with their predicted biological function, associated with plant responses to rehydration upon water stress (WS), found differentially expressed between treatments [control (C)-optimal irrigation (OI)+OI, *Trichoderma simmonsii* T137 (T137)-OI+OI, C-WS+OI, T137-WS+OI] from the recovery experiment. Values correspond to Log_2_ Fold change values and significance is indicated by * (*p* adjusted value < 0.05).

Supplementary Figure 1 Phenotype of *Trichoderma* (T25 and T137)-treated or untreated (Control, C) wheat plants at sampling time, subjected to optimal irrigation (OI), water stress (WS) and rehydration upon WS (WS+OI) conditions. WS experiment (**A** and **B**). Recovery experiment (**C** and **D**).

Supplementary Figure 2 T-distributed Stochastic Neighbour Embedding (tSNE) of all samples from water stress and recovery experiments.

Supplementary Figure 3 Number of DEGs obtained from key comparisons in both water stress **(A)** and recovery **(B)** experiments. Two log_2_ FoldChange (LFC) thresholds were applied, with *|*LFC*|* > 1, and *|*LFC*|* > 2.

Supplementary Figure 4 Number of DEGs corresponding to each wheat chromosome from key comparison of water stress experiment **(A)** and recovery experiment **(B)**. Blue bars correspond to upregulated genes, yellow bars to downregulated genes, and green bars to overall set of genes showing differential expression. U0: DEGs located on the unknown wheat chromosome (version 2.1).

Supplementary Figure 5 DEGs manually annotated with drought related GO (biological process). Key comparison from the water stress experiment (**A**). Key comparison from the recovery experiment (**B**).
