# Supplementary material for: Transcriptomic and biochemical analyses reveal wheat drought mitigation by Trichoderma simmonsii and reduced demand for canonical plant stress responses
Source: Front Plant Sci. 2025 Nov 17;16:1716657. doi: 10.3389/fpls.2025.1716657 (PMC12666693; doi:10.3389/fpls.2025.1716657)
Supplement: Supplementary Table 1 — Primer pairs used for quantitative real time PCR, with forward and reverse sequences and gene name with sources. [file Table1.docx]

## **Table S1.** Primer pairs used for quantitative real time PCR.

| **Primer** | **Sequence (5’-3’)** | **Gene** | **Source** |
| --- | --- | --- | --- |
| WZY2-Fw | GGCAGCTCTAGTAGCTCCAG | *WZY2* | This study |
| WZY2-Rv | CTGTCCGTAGGTGGTGTCAG |  |  |
| DHN16-Fw | TACGGACAGCAAGGTCATAC | *DHN16* | Illescas et al. (2022) |
| DHN16-Rv | TCCATGATGCCCTTCTTCTC |  |  |
| P5CR-Fw | TGGCTGATGGTGGAGTTG | *P5CR* | Illescas et al. (2022) |
| P5CR-Rv | GCTGCCCTGGATGTTTAC |  |  |
| LOX1-Fw | TCCGAGTTCCTGCTCAAGACC | *LOX1* | Risoli et al. (2023) |
| LOX1-Rv | GGCGAAGAAGACACGGCTGT |  |  |
| GRAS-Fw | CGACAACACATTGTGAAGGAAC | *GRAS* | This study |
| GRAS-Rv | AGCACCTAGACGCTGGATTG |  |  |
| WRKY-Fw | GGAACAGCGTCCATTCCATTAC | *WRKY* | This study |
| WRKY-Rv | ACGTGCAGGTCAAGGCTCAG |  |  |
| NAC-Fw | GCGGATCCCTTCAGTCTGTC | *NAC* | This study |
| NAC-Rv | CAGAGCTGCTCGTAACCATCC |  |  |
| ERF-Fw | GACCTGAGCCTGGCGATG | ERF | This study |
| ERF-Rv | CGGTGTCGCTCTGCTCTTC |  |  |
| ACT-Fw | TGACCGTATGAGCAAGGAG | *ACTIN* | Rubio et al. (2019) |
| ACT-Rv | CCAGACAACTCGCAACTTAG |  |  |
