# Supplementary material for: Transcriptomic and biochemical analyses reveal wheat drought mitigation by Trichoderma simmonsii and reduced demand for canonical plant stress responses
Source: Front Plant Sci. 2025 Nov 17;16:1716657. doi: 10.3389/fpls.2025.1716657 (PMC12666693; doi:10.3389/fpls.2025.1716657)
Supplement: Supplementary Table 2 — Wheat gene summary with their symbol and ID (version 2.1), and putative biological function, associated with plant responses to water stress (WS), found differentially expressed between treatments [control (C)-optimal irrigation (OI), Trichoderma simmonsii T137 (T137)-OI, C-WS, T137-WS] from the WS experiment. Values correspond to Log2 Fold change values and significance is indicated by * (p adjusted value < 0.05). [file Table2.docx]

**Table S2**. Wheat gene summary with their symbol and ID (version 2.1), and putative biological function, associated with plant responses to water stress (WS), found differentially expressed between treatments [control (C)-optimal irrigation (OI), *Trichoderma simmonsii* T137 (T137)-OI, C-WS, T137-WS] from the WS experiment. Values correspond to Log_2_ Fold change values and significance is indicated by * (*p* adjusted value < 0.05).

| **Function**** | **Gene symbol** | **Gene ID** | **T137-OI vs C-OI** | **C-WS vs C-OI** | **T137-WS vs C-OI** | **T137-WS vs C-WS** | | **KEGG** |
| --- | --- | --- | --- | --- | --- | --- | --- | --- |
| Antioxidants | *GPX* | TraesCS2D03G1196600 | 0.19 | 2.29 (*) | 1.91 (*) | | -0.41 | K00432 |
|  | *GPX* | TraesCS6A03G0669900 | -0.17 | 2.36 (*) | 1.09 (*) | | -1.30 | K00432 |
|  | *GPX* | TraesCS6D03G0559900 | 0.11 | 2.25 (*) | 1.49 (*) | | -0.78 | K00432 |
|  | *GPX* | TraesCS2B03G1093200 | -0.09 | 1.24 (*) | 1.42 (*) | | 0.16 | K00432 |
|  | *SOD2* | TraesCS2A03G1241200 | 0.33 | 2.26 (*) | 2.00 (*) | | -0.28 | K04564 |
|  | *SOD2* | TraesCS4A03G0974600 | -0.26 | -2.52 (*) | -1.06 | | 1.46 | K04564 |
|  | *SOD1* | TraesCS2D03G0260200 | 0.16 | 1.97 (*) | 1.30 (*) | | -0.69 | K04565 |
|  | *SOD1* | TraesCS2A03G0249400 | -0.05 | 1.96 (*) | 1.16 (*) | | -0.81 | K04565 |
|  | *CAT* | TraesCS6A03G0093800 | 1.35 (*) | 1.97 (*) | 2.68 (*) | | 0.70 | K03781 |
|  | *CAT* | TraesCS6D03G0101500 | 1.08 (*) | 2.40 (*) | 2.21 (*) | | -0.21 | K03781 |
|  | *CAT* | TraesCS6B03G0133900 | 0.63 | 2.25 (*) | 2.51 (*) | | 0.24 | K03781 |
|  | *POX* | TraesCS7A03G1097700 | -0.53 | -11.91 (*) | -2.75 (*) | | 9.15 (*) | K00430 |
|  | *POX* | TraesCS6D03G0113300 | -0.13 | -11.53 (*) | -2.44 (*) | | 9.07 (*) | K00430 |
|  | *POX* | TraesCS6A03G0106600 | -0.19 | -6.64 (*) | -1.94 | | 4.74 (*) | K00430 |
|  | *POX* | TraesCS5A03G0246400 | 0.37 | -7.43 (*) | -2.59 (*) | | 4.72 (*) | K00430 |
|  | *POX* | TraesCS2D03G0807700 | NA | 8.56 (*) | 7.85 (*) | | -0.74 | K00430 |
|  | *POX* | TraesCS3D03G0695400 | NA | 5.36 (*) | 4.40 (*) | | -0.96 | K00430 |
|  | *APX* | TraesCS2A03G1039900 | 0.33 | -3.55 (*) | -1.74 (*) | | 1.80 (*) | K00434 |
|  | *APX* | TraesCS2D03G0157900 | -0.08 | -1.38 (*) | -0.38 (*) | | 0.98 | K00434 |
|  | *APX* | TraesCS2A03G0160200 | -0.04 | -1.23 | -0.10 | | 1.12 | K00434 |
|  | *APX* | TraesCS2B03G0223300 | -0.12 | -1.80 (*) | -0.51 | | 1.27 | K00434 |
| PHP | *PAL* | TraesCS2B03G0540500 | 4.40 | 3.84 | 6.05 (*) | | 2.25 (*) | K10775 |
|  | *PAL* | TraesCS1D03G0079200 | 0.01 | -8.27 (*) | -0.73 | | 7.52 (*) | K10775 |
|  | *PAL* | TraesCS2A03G0409200 | NA | NA | 2.20 | | 7.44 (*) | K10775 |
|  | *PAL* | TraesCS2D03G0427700 | 1.15 | -0.82 | 1.90 (*) | | 2.71 (*) | K10775 |
|  | *PAL* | TraesCS1B03G0334900 | 2.48 | 3.65 (*) | 4.87 (*) | | 1.17 | K10775 |
|  | *PAL* | TraesCS2A03G0409800 | 2.09 | 3.11 | 4.90 (*) | | 1.79 | K10775 |
|  | *CHS* | TraesCS2B03G0101800 | -0.24 | -2.67 (*) | -1.99 (*) | | 0.66 | K00660 |
|  | *CHS* | TraesCS2D03G0053400 | -0.54 | -2.98 (*) | -1.92 | | 1.06 | K00660 |
|  | *CHS* | TraesCS1B03G0082200 | NA | 1.81 | 2.33 (*) | | 0.50 | K00660 |
|  | *CHS* | TraesCS6B03G0471600 | 0.56 | -7.64 (*) | -2.67 | | NA | K00660 |
|  | *CHS* | TraesCS2B03G0080400 | -0.11 | -1.73 (*) | -1.14 (*) | | 0.58 | K00660 |
|  | *CHI* | TraesCS5B03G1189000 | 0.11 | -2.66 (*) | -1.67 (*) | | 0.98 | K01859 |
|  | *CHI* | TraesCS5A03G1120900 | 0.22 | -2.56 (*) | -1.82 (*) | | 0.73 | K01859 |
|  | *CHI* | TraesCS5D03G0364800 | -0.17 | -3.29 (*) | -1.94 (*) | | 1.34 | K01859 |
|  | *FLS* | TraesCS3D03G0769200 | NA | 8.88 | 9.76 (*) | | 0.85 | K05278 |
|  | *FLS* | TraesCS3A03G0837700 | NA | 6.27 | 8.01 (*) | | 1.71 | K05278 |
|  | *FLS* | TraesCS4A03G1102500 | -0.18 | -8.89 (*) | -1.99 (*) | | 6.77 (*) | K05278 |
|  | *FLS* | TraesCS1B03G0233100 | -2.06 | -7.72 (*) | 0.12 | | 7.82 (*) | K05278 |
|  | *FLS*  *FLS* | TraesCS3B03G0961300  TraesCS2B03G1425100 | NA  NA | NA  7.1 (*) | 8.34 (*)  NA | | 5.90  -7.45 (*) | K05278  K05278 |
|  | *FLS* | TraesCS3D03G0887800 | -0.02 | 3.24 (*) | 1.57 (*) | | -2.17 (*) | K05278 |
|  | *FLS* | TraesCS3B03G1088500 | -1.6 | 3.48 (*) | 2.22 (*) | | -1.28 (*) | K05278 |
|  | *FLS* | TraesCS4B03G0902300 | 0.4 | -2.19 (*) | 0.01 | | 2.18 (*) | K05278 |
|  | *DFR* | TraesCS3D03G0544800 | -0.59 | 3.82 (*) | 3.21 (*) | | -0.63 | K13082 |
|  | *DFR* | TraesCS2D03G0388700 | -0.01 | 1.99 (*) | 1.01 (*) | | -0.99 | K13082 |
|  | *DFR* | TraesCS7D03G1043300 | -0.15 | 3.01 (*) | 2.26 | | -0.76 | K13082 |
|  | *ANS* | TraesCS6D03G0011000 | NA | 6.98 (*) | 6.24 (*) | | -0.72 | K05277 |
|  | *ANS* | TraesCS6A03G0003500 | NA | 7.73 (*) | 7.02 | | -0.72 | K05277 |
|  | *ANS* | TraesCS6A03G0094400 | NA | 5.16 (*) | 5.14 (*) | | -0.4 | K05277 |
| AQP | *AQP-SIP* | TraesCS4D03G0446700 | 0.05 | -1.83 (*) | -1.38 (*) | | 0.43 | K09875 |
|  | *AQP-SIP* | TraesCS4A03G0266800 | -0.06 | -1.84 (*) | -2.24 (*) | | 0.57 | K09875 |
|  | *AQP-SIP* | TraesCS4B03G0500200 | -0.08 | -1.84 (*) | -0.88 (*) | | 0.94 | K09875 |
|  | *AQP-NIP* | TraesCS1A03G0479500 | 0.02 | -7.21 (*) | -4.54 (*) | | NA | K09874 |
|  | *AQP-NIP* | TraesCS5A03G0822900 | 0.18 | -6.15 (*) | -2.84 (*) | | 3.19 | K09874 |
|  | *AQP-NIP* | TraesCS7A03G0501900 | 1.34 | -2.82 (*) | -2.62 (*) | | 0.17 | K09874 |
|  | *AQP-NIP* | TraesCS7D03G0484800 | 1.15 | -2.54 (*) | -2.05 (*) | | 0.48 | K09874 |
|  | *AQP-PIP* | TraesCS5A03G0802100 | 0.10 | -5.17 (*) | -1.46 (*) | | 3.69 (*) | K09872 |
|  | *AQP-PIP* | TraesCS5B03G0835100 | 0.19 | -5.83 (*) | -2.57 (*) | | 3.26 (*) | K09872 |
|  | *AQP-PIP* | TraesCS5D03G0759700 | 0.23 | -3.79 (*) | -1.09 (*) | | 2.69 (*) | K09872 |
|  | *AQP-PIP* | TraesCS2D03G0860200 | -0.26 | -3.74 (*) | -1.28 (*) | | 2.44 (*) | K09872 |
|  | *AQP-TIP* | TraesCS3D03G1192600 | -0.03 | -5.88 (*) | -2.85 (*) | | 3.01 (*) | K09873 |
|  | *AQP-TIP* | TraesCS3B03G1515200 | 0.00 | -4.90 (*) | -2.34 (*) | | 2.55 (*) | K09873 |
|  | *AQP-TIP* | TraesCS2B03G0998700 | -0.40 | 7.03 (*) | 5.13 (*) | | -1.91 (*) | K09873 |
|  | *AQP-TIP* | TraesCS1A03G0448700 | 0.18 | 3.44 (*) | -2.03 | | -5.49 (*) | K09873 |
| Drought-response | *HSP* | TraesCS3B03G0851600 | 0.01 | -2.47 (*) | -0.73 | | 1.73 (*) | K11940 |
|  | *HSP* | TraesCS3D03G0687100 | 0.12 | -1.14 (*) | -0.21 | | 0.92 (*) | K11940 |
|  | *HSP* | TraesCS6A03G0882200 | -0.39 | -8.64 (*) | -2.48 | | NA | K03283 |
|  | *HSP* | TraesCS4A03G0203800 | -0.31 | 8.63 (*) | 6.43 (*) | | -2.23 (*) | K03283 |
|  | *HSP* | TraesCS4D03G0504900 | 0.18 | 7.81 (*) | 5.71 (*) | | -2.12 (*) | K03283 |
|  | *LEA* | TraesCS2B03G1193200 | -0.69 | 8.25 (*) | 4.51 (*) | | -3.77 (*) | NA |
|  | *LEA* | TraesCS1A03G0887200 | -0.97 | 9.20 (*) | 5.39 (*) | | -3.84 (*) | NA |
|  | *LEA* | TraesCS2D03G0807600 | 0.15 | 2.15 (*) | 1.10 (*) | | -1.07 (*) | NA |
|  | *DHN* | TraesCS6D03G0772500 | -0.58 | 9.16 (*) | 6.60 (*) | | -2.59 (*) | NA |
|  | *DHN* | TraesCS5B03G0937300 | -0.71 | 9.22 (*) | 6.73 (*) | | -2.52 | NA |
|  | *DHN* | TraesCS6B03G1083400 | -0.81 | 8.97 (*) | 6.15 | | -2.85 | NA |
|  | *DHN* | TraesCS6B03G1084600 | -0.63 | 9.09 (*) | 6.66 (*) | | -2.46 | NA |
|  | *TPS* | TraesCS5A03G0527400 | 0.87 | 2.84 (*) | 2.85 (*) | | -0.01 | K16055 |
|  | *TPS* | TraesCS1D03G0804800 | 0.5 | 1.03 (*) | 1.91 (*) | | 0.85 | K16055 |
|  | *TPS* | TraesCS6A03G0903100 | 0.51 | 2.52 (*) | 1.5 (*) | | -1.03 | K16055 |
|  | *TPS* | TraesCS6B03G1087500 | 0.45 | 2.62 (*) | 1.31 (*) | | -1.34 (*) | K16055 |
|  | *TPS* | TraesCS6D03G0775000 | 0.2 | 1.54 (*) | 0.5 | | -1.05 (*) | K16055 |
|  | *TPP* | TraesCS5A03G0508000 | 0.06 | 0.65 (*) | 1.76 (*) | | 1.09 (*) | K01087 |
|  | *TPP* | TraesCS6A03G0673800 | -0.24 | 1.1 | 2.98 (*) | | 1.85 | K01087 |
|  | *TPP* | TraesCS6D03G0564500 | 0.28 | 0.98 (*) | 1.83 (*) | | 0.83 | K01087 |
|  | *TPP* | TraesCS6B03G0801800 | 0.08 | 0.33 | 1.79 (*) | | 1.45 | K01087 |
| Glycine metabolism | *BADH* | TraesCS6D03G0819500 | 0.21 | 3.21 (*) | 3.37 (*) | | 0.13 | K00130 |
|  | *BADH* | TraesCS6B03G1151100 | 0.39 | 2.9 (*) | 3.18 (*) | | 0.26 | K00130 |
|  | *BADH* | TraesCS2A03G0811300 | -0.01 | 3.27 (*) | 2.88 (*) | | -0.41 | K00130 |
|  | *BADH* | TraesCS2D03G0751400 | 0.05 | 2.94 (*) | 2.64 (*) | | -0.32 | K00130 |
|  | *CMO* | TraesCS7B03G0869300 | -0.36 | -1.1 (*) | -0.78 | | 0.3 | K00499 |
|  | *CMO* | TraesCS7A03G1032000 | 0.06 | -1.14 (*) | -0.21 | | 0.9 | K00499 |
| PA | *P5CS* | TraesCS3D03G0793300 | -0.22 | 8.51 (*) | 7.75 (*) | | -0.79 | K12657 |
|  | *P5CS* | TraesCS1D03G0675000 | 0.35 | 5.02 (*) | 4.71 (*) | | -0.34 | K12657 |
|  | *P5CR* | TraesCS3B03G1339500 | -0.07 | 4.58 (*) | 3.10 (*) | | -1.5 | K00286 |
|  | *P5CR* | TraesCS3D03G1069300 | 0.16 | 4.15 (*) | 2.89 (*) | | -1.29 | K00286 |
|  | *arg* | TraesCS2B03G0102100 | -0.06 | 2.37 (*) | 1.15 (*) | | -1.24 (*) | K01476 |
|  | *OAT* | TraesCS5A03G0900400 | -0.27 | 4.22 (*) | 2.12 (*) | | -2.12 (*) | K00819 |
|  | *ODC1* | TraesCS5A03G0811400 | NA | 7.99 (*) | 6.87 (*) | | -1.13 | K01581 |
|  | *speE* | TraesCS7B03G0654200 | -0.07 | 1.19 (*) | 1.99 (*) | | 0.78 | K00797 |
|  | *speE* | TraesCS7D03G0601900 | -0.35 | 1.85 (*) | 0.52 | | -1.34 | K00797 |
|  | *speE* | TraesCS7A03G0856200 | -0.24 | 1.22 (*) | 1.78 (*) | | 0.54 | K00797 |
|  | *PAO* | TraesCS2A03G1099800 | -0.59 | 0.61 | 1.19 (*) | | 0.56 | K17839 |
|  | *PAO* | TraesCS2D03G1042800 | -0.54 | 0.76 | 1.28 (*) | | 0.5 | K17839 |
| ABA | *NCED* | TraesCS2D03G0580300 | NA | 8.22 (*) | 7.31 (*) | | -0.93 | K09840 |
|  | *NCED* | TraesCS5B03G0943700 | 0.14 | 5.86 (*) | 5.12 (*) | | -0.76 | K09840 |
|  | *NCED* | TraesCS5D03G0859900 | 0.90 | 5.69 (*) | 4.62 (*) | | -1.08 | K09840 |
|  | *ABA2* | TraesCS2A03G0254300 | -0.21 | 6.87 (*) | 5.37 (*) | | -1.53 (*) | K09841 |
|  | *ABA2* | TraesCS4A03G0236300 | NA | 5.33 (*) | 4.81 | | -0.60 | K09841 |
|  | *AAO* | TraesCS5A03G1012300 | 6.72 | 8.76 (*) | 8.18 (*) | | -0.59 | K09842 |
|  | *AAO* | TraesCS5B03G1058500 | -0.12 | 1.41 (*) | 1.00 (*) | | -0.43 | K09842 |
| ET | *ACS* | TraesCS3D03G0223900 | NA | 5.24 (*) | 5.42 (*) | | 0.17 | K01762 |
|  | *ACS* | TraesCS2D03G0897400 | 0.49 | 3.89 (*) | 4.80 (*) | | 0.89 | K20772 |
|  | *ACO* | TraesCS3A03G0775800 | NA | 4.74 (*) | 1.50 | | -3.28 | K05933 |
|  | *ACO* | TraesCS6D03G0712700 | -0.54 | 4.08 (*) | 2.56 (*) | | -1.53 | K05933 |
|  | *ACO* | TraesCS1A03G0217700 | -0.06 | 2.94 (*) | 1.99 (*) | | -0.97 | K05933 |
|  | *ACO* | TraesCS5B03G0609200 | 0.26 | 2.92 (*) | 1.93 (*) | | 0.03 | K05933 |
|  | *EIN2* | TraesCS4B03G0083200 | -0.02 | 1.68 (*) | 2.78 (*) | | 1.08 | K14513 |
|  | *EIN2* | TraesCS4D03G0060200 | 0.66 | 0.03 | 1.36 (*) | | 1.31 (*) | K14513 |
| JA | *LOX* | TraesCS6A03G0314500 | NA | NA | 13.10 (*) | | 13.36 (*) | K15718 |
|  | *LOX* | TraesCS5B03G0014800 | -0.06 | -2.13 | 3.02 (*) | | 5.14 (*) | K00454 |
|  | *LOX* | TraesCS5D03G0028900 | 0.10 | 3.96 (*) | 4.52 (*) | | 4.52 (*) | K00454 |
|  | *LOX* | TraesCS5A03G0017800 | 0.00 | -0.62 | 3.79 (*) | | 4.40 (*) | K00454 |
|  | *LOX* | TraesCS2A03G1219200 | 0.12 | 0.36 | 3.84 (*) | | 3.47 (*) | K15718 |
|  | *LOX* | TraesCS6D03G0343800 | -0.21 | -3.60 (*) | -0.20 | | 3.38 (*) | K00454 |
|  | *LOX* | TraesCS5D03G0104900 | 0.75 | -7.95 (*) | -4.61 (*) | | 3.37 (*) | K00454 |
|  | *LOX* | TraesCS6B03G0497600 | -0.14 | -1.83 (*) | 0.37 | | 2.19 (*) | K00454 |
|  | *AOS* | TraesCS4B03G0647300 | -1.11 | 2.19 | 5.47 (*) | | 3.24 (*) | K01723 |
|  | *AOS* | TraesCS4D03G0575900 | -0.24 | 0.83 | 3.58 (*) | | 2.72 (*) | K01723 |
|  | *AOS* | TraesCS4B03G0647900 | 0.00 | 3.17 (*) | 5.53 (*) | | 2.34 (*) | K01723 |
|  | *AOS* | TraesCS4D03G0576000 | 0.37 | 0.52 | 2.87 (*) | | 2.33 (*) | K01723 |
|  | *AOS* | TraesCS4A03G0122800 | -0.32 | 3.14 (*) | 4.48 (*) | | 1.32 (*) | K01723 |
|  | *AOC* | TraesCS6B03G1030800 | -0.70 | -0.22 | 1.13 (*) | | 1.33 (*) | K10525 |
|  | *AOC* | TraesCS6D03G0731500 | -0.77 | -0.13 | 1.00 (*) | | 1.12 (*) | K10525 |
|  | *AOC* | TraesCS6A03G0861500 | -0.16 | 1.25 (*) | 2.03 (*) | | 0.76 | K10525 |
|  | *JAZ* | TraesCS2A03G0715200 | NA | 3.87 | 6.92 (*) | | 3.08 (*) | K13464 |
|  | *JAZ* | TraesCS7A03G0474000 | NA | 5.08 (*) | 7.85 (*) | | 2.58 (*) | K13464 |
|  | *JAZ* | TraesCS7D03G0457300 | NA | 5.28 (*) | 7.89 (*) | | 2.58 (*) | K13464 |
|  | *JAZ* | TraesCS5D03G0513000 | -1.72 | 0.65 | 3.12 (*) | | 2.45 (*) | K13464 |
| GA | *GA20ox* | TraesCS7B03G1273600 | NA | 5.76 (*) | 4.67 (*) | | -1.17 | K05282 |
|  | *GA20ox* | TraesCS5D03G1210400 | 0.04 | -7.32 (*) | -2.96 (*) | | 4.21 | K05282 |
|  | *GA20ox* | TraesCS5B03G1356500 | 1.03 | -8.13 (*) | -1.27 | | 6.85 (*) | K05282 |
|  | *GA20ox* | TraesCS1D03G0635700 | -0.48 | -7.94 (*) | -1.94 | | NA | K05282 |
|  | *GA13ox* | TraesCS4D03G0432400 | -0.09 | -1.33 (*) | -0.46 | | 0.86 | K20666 |
|  | *GA13ox* | TraesCS4B03G0484900 | 0.34 | -3.80 (*) | -0.75 | | 3.03 | K20666 |
|  | *GID1* | TraesCS1B03G0747800 | -0.17 | -8.23 | 0.95 | | 9.17 (*) | K14493 |
|  | *GID1* | TraesCS1D03G0617800 | 0.32 | 0.85 (*) | 3.52 (*) | | 2.65 (*) | K14493 |
|  | *GID1* | TraesCS1B03G0749400 | -0.20 | 1.20 | 3.31 (*) | | 2.09 (*) | K14493 |
| IAA | *AMI1* | TraesCS2A03G1357400 | -0.02 | -1.39 (*) | -0.78 (*) | | 0.60 | K01426 |
|  | *AMI1* | TraesCS2B03G0077300 | 0.22 | -5.44 (*) | -1.47 | | NA | K01426 |
|  | *AMI1* | TraesCS2D03G0050400 | -0.55 | -7.37 (*) | -1.98 | | NA | K01426 |
|  | *YUCCA* | TraesCS5D03G0526200 | -0.25 | -4.28 (*) | -1.86 (*) | | 2.38 | K11816 |
|  | *YUCCA* | TraesCS4A03G0782000 | -0.42 | -7.26 (*) | -1.57 | | NA | K11816 |
|  | *YUCCA* | TraesCS5B03G1370600 | -0.36 | -8.88 (*) | -1.16 | | 7.71 | K11816 |
|  | *TAA1* | TraesCS3D03G0184300 | -0.21 | -9.82 (*) | -1.48 (*) | | 8.33 (*) | K16903 |
|  | *TAA1* | TraesCS3B03G0713100 | -0.18 | -3.11 (*) | -1.71 (*) | | 1.38 | K16903 |
|  | *TAA1* | TraesCS3B03G0248400 | -0.78 | -5.91 (*) | -2.74 (*) | | 3.14 | K16903 |
|  | *TAA1* | TraesCS3D03G0580800 | -0.06 | -4.62 (*) | -2.58 (*) | | 2.02 | K16903 |
|  | *IAA* | TraesCS5B03G0146300 | 0.39 | -3.70 (*) | -0.70 | | 3.00 (*) | K14484 |
|  | *IAA* | TraesCS5D03G0169600 | 0.19 | -3.05 (*) | -0.76 | | 2.28 (*) | K14484 |
|  | *SAUR* | TraesCS7D03G0167400 | 1.35 | -7.91 (*) | -1.88 | | 5.91 (*) | K14488 |
|  | *SAUR* | TraesCS7B03G0778400 | -0.34 | 2.45 (*) | 0.48 | | -1.98 (*) | K14488 |
| SL | *DWARF27* | TraesCS7D03G0971000 | -0.18 | -8.64 (*) | -1.22 | | 7.40 (*) | K17911 |
|  | *DWARF27* | TraesCS7B03G0855500 | -0.15 | -6.30 (*) | -4.13 (*) | | 2.11 | K17911 |
|  | *DWARF27* | TraesCS1D03G0173800 | 0.14 | -1.96 (*) | -1.05 | | 0.90 | K17911 |
|  | *CCD* | TraesCS5B03G0003200 | -0.19 | -1.53 (*) | -0.92 (*) | | 0.59 | K00465 |
|  | *CCD* | TraesCS5D03G0005200 | 0.48 | -8.33 (*) | -2.02 | | 6.30 | K00465 |
|  | *CYP711A1* | TraesCS4A03G1019000 | NA | 2.56 | NA | | -7.57 (*) | K20771 |
|  | *CYP711A1* | TraesCS6A03G0470400 | 0.04 | -4.70 (*) | -3.43 (*) | | 1.29 | K20771 |
|  | *CYP711A1* | TraesCS6D03G0392500 | 0.17 | -8.60 (*) | -3.62 (*) | | NA | K20771 |
| CK | *CYP735A* | TraesCS7B03G0638400 | 0.24 | -9.10 (*) | -3.00 (*) | | NA | K10717 |
|  | *CRE1* | TraesCS4D03G0091900 | 0.13 | -2.71 (*) | -0.88 (*) | | 1.81 (*) | K14489 |
| BR | *90A-1* | TraesCS5D03G0351000 | 0.09 | -0.90 (*) | -1.00 (*) | | -0.12 | K09588 |
|  | *90A-1* | TraesCS5A03G0368600 | 0.42 | -0.90 (*) | -1.16 (*) | | -0.47 | K09588 |
|  | *DET2* | TraesCS3D03G0796800 | 0.35 | -2.42 (*) | -0.82 | | 1.58 | K09591 |
|  | *DET2* | TraesCS3A03G0869900 | 0.14 | -8.60 (*) | -1.98 (*) | | 6.61 (*) | K09591 |
|  | *CYP92A6* | TraesCS5B03G0168300 | 0.30 | -8.90 (*) | -0.62 | | 8.26 (*) | K20623 |
|  | *CYP92A6* | TraesCS5A03G0165800 | -0.52 | -3.52 (*) | 0.52 | | 4.02 (*) | K20623 |
|  | *CYP92A6* | TraesCS5D03G0509300 | 0.19 | -1.55 (*) | 0.51 | | 2.04 (*) | K20623 |
|  | *CYP92A6* | TraesCS5D03G0509500 | 0.18 | 0.96 (*) | 2.40 (*) | | 1.42 (*) | K20623 |
|  | *CYP92A6* | TraesCS5B03G0555700 | 0.36 | 0.30 | 1.66 (*) | | 1.35 (*) | K20623 |
|  | *CYP92A6* | TraesCS5D03G0181600 | -0.42 | -0.73 | 1.83 (*) | | 2.54 | K20623 |
|  | *CYP85A1* | TraesCS2D03G0028100 | 0.05 | -7.19 (*) | -3.74 | | NA | K09590 |
|  | *CYP85A1* | TraesCS2D03G0027500 | -0.48 | -8.23 (*) | -3.48 | | NA | K09590 |
|  | *BRI1* | TraesCS7A03G1324900 | 0.10 | -8.49 (*) | -2.61 (*) | | 5.99 (*) | K13415 |
|  | *BRI1* | TraesCS3B03G1371800 | 0.01 | -4.22 (*) | -0.81 | | 3.40 (*) | K13415 |
|  | *BRI1* | TraesCS2D03G0350600 | 0.16 | -2.09 (*) | 0.09 | | 2.17 (*) | K13415 |
| Signal  transduction | *CDPK* | TraesCS2A03G0416900 | 0.42 | 0.16 | 1.41 (*) | | 1.23 (*) | K13412 |
|  | *CDPK* | TraesCS6A03G0203200 | 0.20 | 0.03 | 1.19 (*) | | 1.15 (*) | K13412 |
|  | *CDPK* | TraesCS7A03G0620400 | NA | 7.87 (*) | 8.19 (*) | | 0.30 | K13412 |
|  | *MAPK* | TraesCS1A03G0953400 | 0.27 | NA | 2.61 | | 8.16 (*) | K20604 |
|  | *MAPK* | TraesCS7A03G0826400 | -0.32 | -2.73 (*) | 1.12 | | 3.83 (*) | K20716 |
|  | *MAPK* | TraesCS1D03G0951400 | -0.33 | -3.31 (*) | 0.38 | | 3.68 (*) | K20538 |
|  | *MAPK* | TraesCS1B03G1166400 | -0.29 | -2.77 (*) | -0.01 | | 2.75 (*) | K20538 |
|  | *MAPK* | TraesCS1A03G0983400 | -0.23 | -1.64 (*) | 1.06 (*) | | 2.68 (*) | K20538 |
|  | *MAPK* | TraesCS1B03G1045300 | NA | 6.85 (*) | 4.17 (*) | | -2.71 (*) | K20716 |
|  | *MAPK* | TraesCS1A03G0892100 | 0.14 | 5.95 (*) | 2.62 (*) | | -3.34 (*) | K20716 |
|  | *PP2C* | TraesCS1A03G1005000 | -0.47 | 5.57 (*) | 3.53 (*) | | -2.07 (*) | K14497 |
|  | *PP2C* | TraesCS1D03G0967400 | NA | 7.36 (*) | 4.65 (*) | | -2.77 (*) | K14497 |
|  | *PP2C* | TraesCS1B03G1186600 | NA | 8.82 (*) | 6.75 (*) | | -2.12 (*) | K14497 |
|  | *LRR-RLK* | TraesCS7A03G0388200 | -0.13 | -8.56 (*) | -1.07 | | 7.48 (*) | K20718 |
|  | *LRR-RLK* | TraesCS7A03G0148800 | 0.56 | -6.76 (*) | -1.21 (*) | | 5.48 (*) | K20718 |
|  | *LRR-RLK* | TraesCS1B03G0018500 | -0.09 | -5.56 (*) | -2.16 (*) | | 3.40 (*) | K20718 |
|  | *LRR-RLK* | TraesCS4A03G1197000 | -1.08 | -1.19 (*) | 0.47 | | 1.64 (*) | K20718 |
| Pathogen-related | *NPR1* | TraesCS3A03G0230900 | -0.10 | 0.42 | 1.71 (*) | | 1.28 | K14508 |
|  | *NPR1* | TraesCS3B03G0287300 | 0.68 | 1.28 (*) | 1.63 (*) | | 0.33 | K14508 |

** PHP: phenylpropanoid; AQP: aquaporin; PA: polyamine; ABA: abscisic acid; ET: ethylene; JA: jasmonic acid; GA: gibberellin; IAA: indole-acetic acid; SL: strigolactone; CK: cytokinin; BR: brasinosteroid; NA: not available.
