# Supplementary material for: Transcriptomic and biochemical analyses reveal wheat drought mitigation by Trichoderma simmonsii and reduced demand for canonical plant stress responses
Source: Front Plant Sci. 2025 Nov 17;16:1716657. doi: 10.3389/fpls.2025.1716657 (PMC12666693; doi:10.3389/fpls.2025.1716657)
Supplement: Supplementary Table 3 — Compilation of wheat genes, including their symbol and corresponding ID (version 2.1), along with their predicted biological function, associated with plant responses to rehydration upon water stress (WS), found differentially expressed between treatments [control (C)-optimal irrigation (OI)+OI, Trichoderma simmonsii T137 (T137)-OI+OI, C-WS+OI, T137-WS+OI] from the recovery experiment. Values correspond to Log2 Fold change values and significance is indicated by * (p adjusted value < 0.05). [file Table3.docx]

**Table S3**. Compilation of wheat genes, including their symbol and corresponding ID (version 2.1), along with their predicted biological function, associated with plant responses to rehydration upon water stress (WS), found differentially expressed between treatments [control (C)-optimal irrigation (OI)+OI, *Trichoderma simmonsii* T137 (T137)-OI+OI, C-WS+OI, T137-WS+OI] from the recovery experiment. Values correspond to Log_2_ Fold change values and significance is indicated by * (*p* adjusted value < 0.05).

| **Function**** | **Gene symbol** | **Gene ID** | **T137-OI+OI vs C-OI+OI** | **C-WS+OI vs C-OI+OI** | **T137-WS+OI vs C-OI+OI** | **T137-WS+OI vs C-WS+OI** | **KEGG** |
| --- | --- | --- | --- | --- | --- | --- | --- |
| Carbohydrate metabolism | *bglB* | TraesCS2A03G0274500 | -0.48 | NA | 0.76 | 6.85 (*) | K05350 |
|  | *AMY* | TraesCS7D03G0895200 | -0.42 | -2.02 (*) | -0.46 | 1.56 (*) | K01176 |
|  | *RafS* | TraesCS3D03G0183600 | 0.23 | -0.16 | 0.97 | 1.13 (*) | K06617 |
|  | *rcbL* | TraesCS3B03G0446200 | 1.72 (*) | -0.22 | 2.22 (*) | 2.44 (*) | K01601 |
|  | *rcbL* | TraesCS5D03G0016000 | 1.81 (*) | 0.16 | 2.47 (*) | 2.30 (*) | K01601 |
|  | *rcbL* | TraesCS5D03G0936400 | 1.02 | -0.70 | 1.28 | 1.97 (*) | K01601 |
| Energy metabolism | *psbH* | TraesCS5D03G1229900 | 1.19 | -1.61 (*) | 0.56 | 2.17 (*) | K02709 |
|  | *ATPF1B* | TraesCS1D03G0339900 | 0.46 | -1.38 (*) | 0.25 | 1.63 (*) | K02112 |
| Lipid  metabolism | *baiN* | TraesCS5D03G0010400 | -0.87 | -8.59 (*) | -1.25 | 7.35 (*) | K07007 |
|  | *CER1* | TraesCS2A03G0932600 | 1.13 | -21.59 | -0.26 | 7.21 (*) | K15404 |
|  | *FAR* | TraesCS3D03G0023400 | NA | NA | 2.67 | 6.85 (*) | K13356 |
|  | *FAR* | TraesCS1D03G0041600 | 0.4 | -1.7 | 0.28 | 2.07 (*) | K13356 |
|  | *KCS* | TraesCS5B03G0022900 | 0.4 | -0.92 | 1.27 | 2.2 (*) | K15397 |
|  | *WSD1* | TraesCS3B03G0017600 | 0.25 | -1.15 | 0.66 | 1.81 (*) | K15406 |
|  | *CYP17A* | TraesCS1D03G0992300 | 0.03 | 2.22 | 0.64 | -1.58 | K00512 |
|  | *LOX1* | TraesCS6A03G0314400 | 0.69 | 1.82 (*) | -3.26 (*) | -5.1 (*) | K15718 |
| Replication  and repair | *XRCC6* | TraesCS5A03G0130000 | 0.92 | -6.44 (*) | 0.23 | 6.67 (*) | K10884 |
|  | *MSH6* | TraesCS3B03G0323600 | 0.37 | NA | 0.96 | 6.81 (*) | K08737 |
| Transport | *ABCG2* | TraesCS5B03G1381800 | 0.76 | -21.41 | 0.15 | 7.45 (*) | K08712 |
|  | *SLC15A3* | TraesCS7B03G0773600 | 0.29 | -6.81 | 0.20 | 7.01 (*) | K14638 |
|  | *SLC2A8* | TraesCS5A03G1196400 | 0.68 | NA | 1.09 | 7.00 (*) | K08145 |
|  | *YME1* | TraesCS4A03G0863700 | 8.38 (*) | 7.31 (*) | 7.74 (*) | 0.4 | K08955 |
| Antioxidants | *POX* | TraesCS4D03G0233800 | NA | NA | 4.07 (*) | 7.25 (*) | K00430 |
|  | *POX* | TraesCS1B03G0970400 | 1.45 | 0.04 | 3.02 (*) | 2.98 (*) | K00430 |
|  | *POX* | TraesCS4A03G0539300 | -0.30 | 0.61 | -0.69 | -1.30 (*) | K00430 |
| AQP | *AQP-PIP* | TraesCS6B03G0761200 | 0.81 | 0.71 | 1.71 (*) | 1.00 (*) | K09872 |
|  | *AQP-NIP* | TraesCS1D03G0452900 | 0.68 | 0.93 | 1.61 (*) | 0.68 | K09874 |
|  | *AQP-PIP* | TraesCS6A03G1015200 | 0.67 | 0.96 | 1.56 (*) | 0.61 | K09872 |
|  | *AQP-TIP* | TraesCS3B03G1515200 | 0.52 | 1.32 (*) | 1.53 | 0.21 | K09873 |
| Signal transduction | *CDPK* | TraesCS5A03G0725600 | -0.96 | -21.33 | 0.53 | 7.74 (*) | K13412 |
|  | *IRAK1* | TraesCS2D03G0038900 | 1.15 | NA | 0.93 | 7.11 (*) | K04730 |
|  | *IRAK1* | TraesCS6D03G0002800 | 7.9 (*) | NA | NA | 1.03 | K04730 |
|  | *QARS* | TraesCS5B03G0210200 | NA | NA | 1.70 | 6.95 (*) | K01886 |
| Translation | *rpsN* | TraesCS2B03G1229000 | 0.41 | -2.63 (*) | -0.33 | 2.30 (*) | K02954 |
|  | *rplV* | TraesCS6D03G0396800 | 1.84 | -0.51 | 0.66 | 1.17 (*) | K02890 |
| Aa metabolism | *mtnN* | TraesCS3B03G0434100 | 1.64 (*) | 1.86 | 7.74 (*) | 0.19 | K01243 |
|  | *ACP7* | TraesCS3B03G0912100 |  |  |  |  | K22390 |
| Unclassified | *NAS* | TraesCS6D03G0330700 | -1.28 | -8.66 (*) | -0.63 | 8.04 (*) | K05953 |
|  | *ruvX* | TraesCS3A03G0746700 | -0.47 | -7.51 (*) | -0.58 | 6.93 (*) | K07447 |

NA: not available
