# Supplementary figures and images for: Transcriptomic and biochemical analyses reveal wheat drought mitigation by Trichoderma simmonsii and reduced demand for canonical plant stress responses
Source: Front Plant Sci. 2025 Nov 17;16:1716657. doi: 10.3389/fpls.2025.1716657 (PMC12666693; doi:10.3389/fpls.2025.1716657)

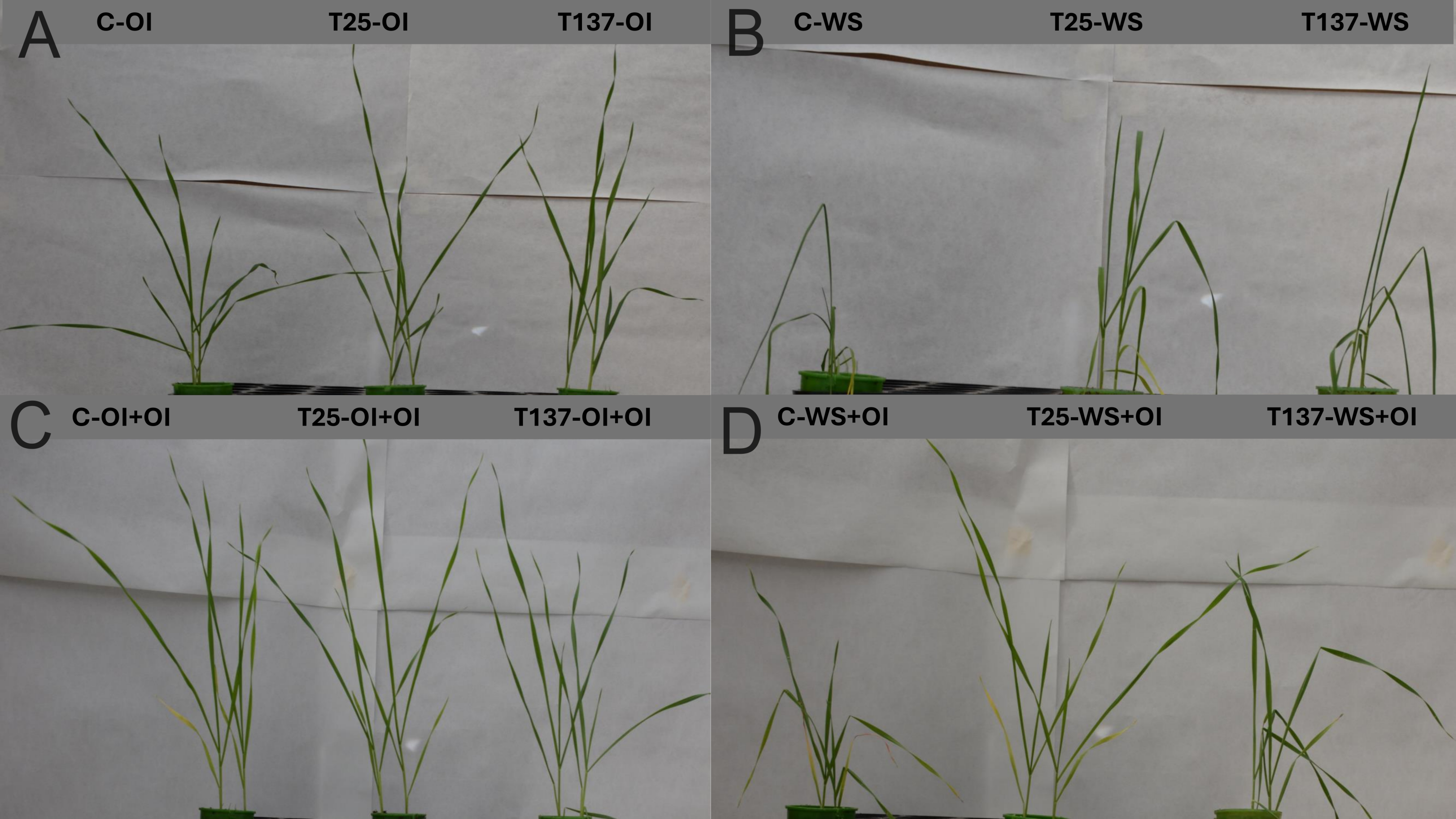

Supplement: Supplementary Figure 1 — Phenotype of Trichoderma (T25 and T137)-treated or untreated (Control, C) wheat plants at sampling time, subjected to optimal irrigation (OI), water stress (WS) and rehydration upon WS (WS+OI) conditions. WS experiment (A and B). Recovery experiment (C and D). [file Image1.jpeg]

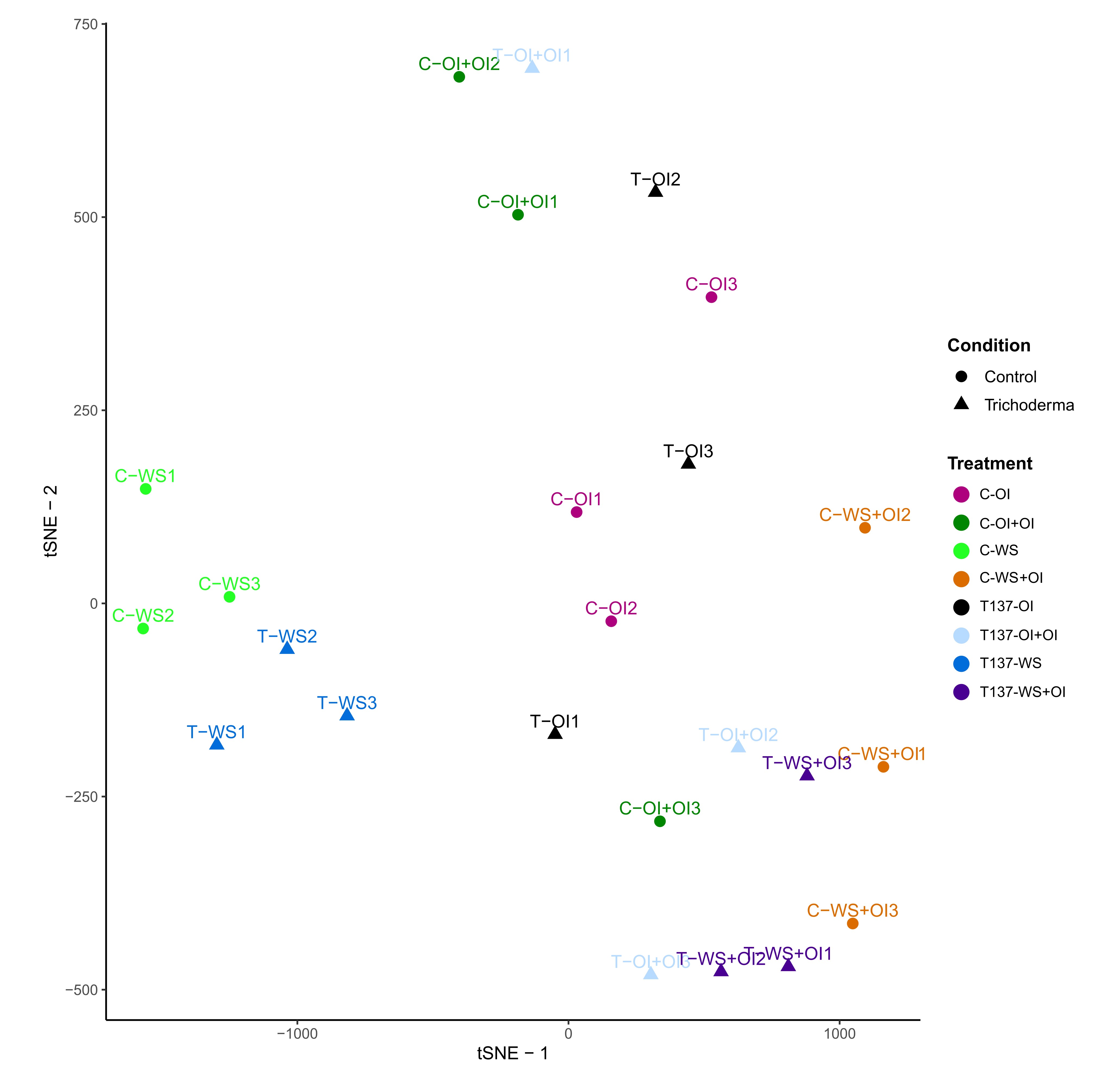

Supplement: Supplementary Figure 2 — T-distributed Stochastic Neighbour Embedding (tSNE) of all samples from water stress and recovery experiments. [file Image2.jpeg]

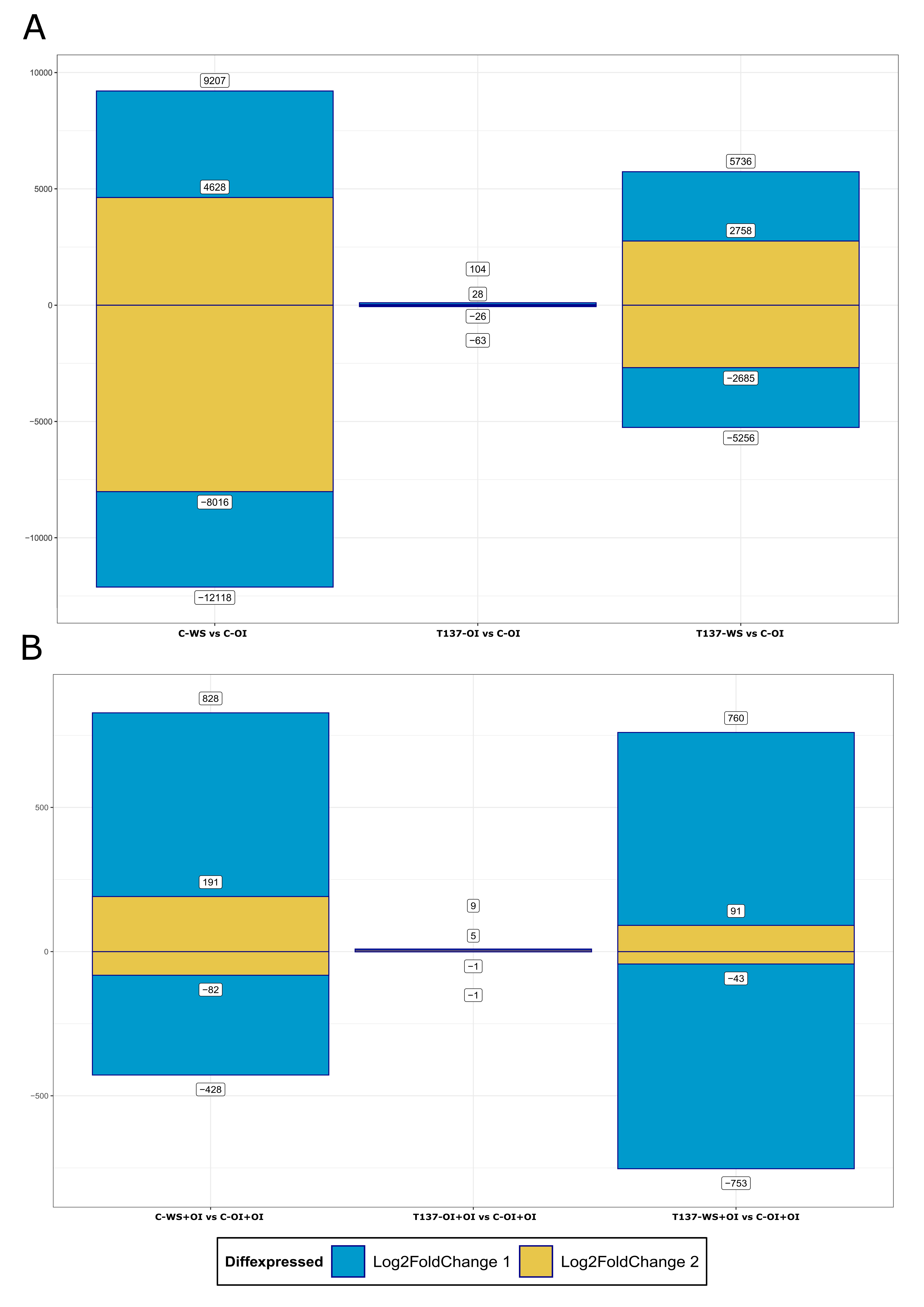

Supplement: Supplementary Figure 3 — Number of DEGs obtained from key comparisons in both water stress (A) and recovery (B) experiments. Two log2 FoldChange (LFC) thresholds were applied, with |LFC| > 1, and |LFC| > 2. [file Image3.jpeg]

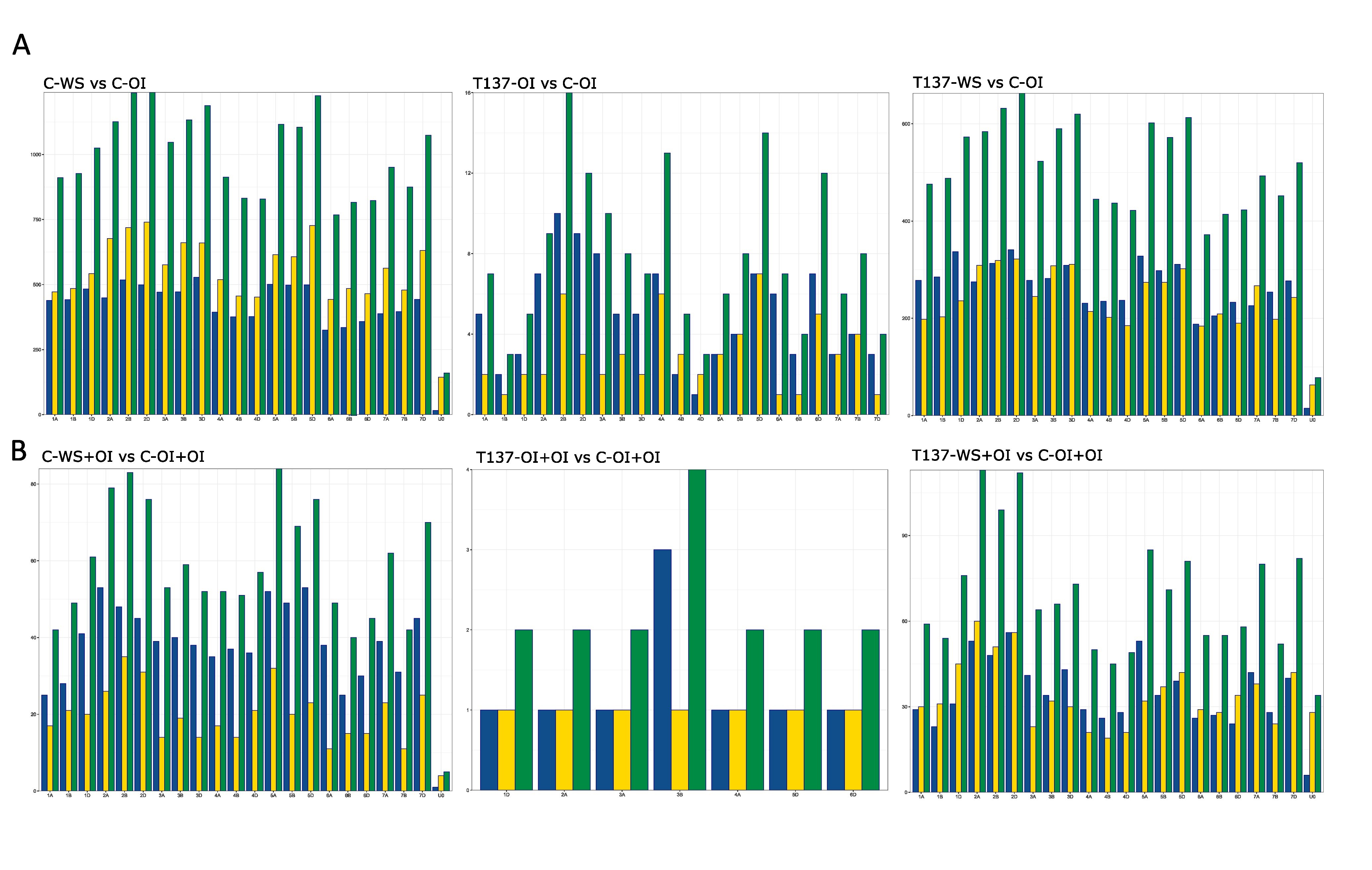

Supplement: Supplementary Figure 4 — Number of DEGs corresponding to each wheat chromosome from key comparison of water stress experiment (A) and recovery experiment (B). Blue bars correspond to upregulated genes, yellow bars to downregulated genes, and green bars to overall set of genes showing differential expression. U0: DEGs located on the unknown wheat chromosome (version 2.1). [file Image4.jpeg]

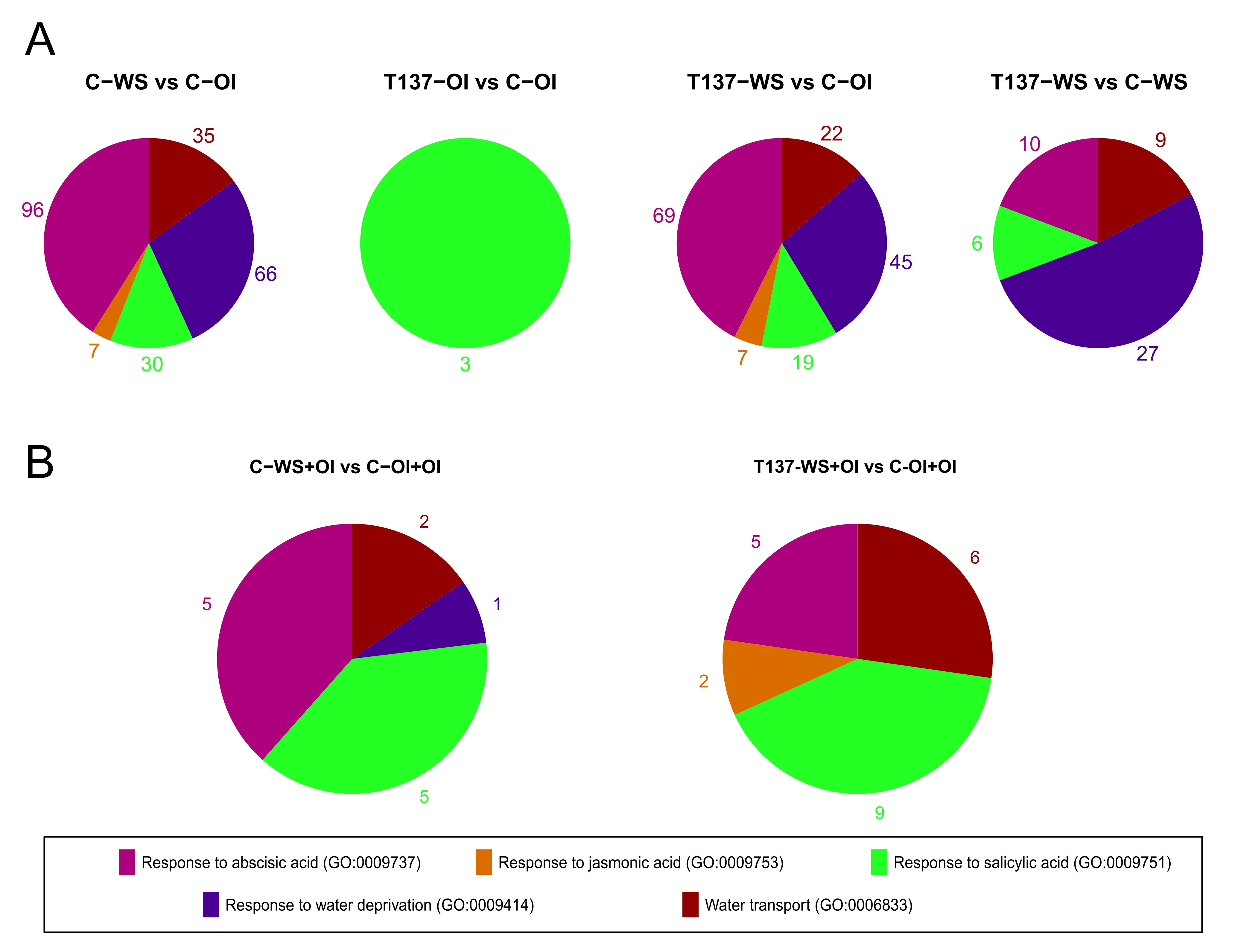

Supplement: Supplementary Figure 5 — DEGs manually annotated with drought related GO (biological process). Key comparison from the water stress experiment (A). Key comparison from the recovery experiment (B). [file Image5.jpeg]
